# Personality and mental health as mediators linking childhood maltreatment to intimate partner violence victimization: a Mendelian randomization–direction of causation twin study

**DOI:** 10.1016/j.lanepe.2026.101653

**Published:** 2026-03-26

**Authors:** Patrizia Pezzoli, Wikus Barkhuizen, Olakunle Oginni, Jean-Baptiste Pingault, Eamon McCrory, Essi Viding

**Affiliations:** a Division of Psychology and Language Sciences, University College London (UCL), UK; b Social, Genetic & Developmental Psychiatry Centre, Institute of Psychiatry, Psychology & Neuroscience, King's College London, UK; c Wolfson Centre for Young People's Mental Health and Centre for Neuropsychiatric Genetics and Genomics, Division of Psychological Medicine and Clinical Neuroscience, Cardiff University, Cardiff, UK

**Keywords:** Childhood maltreatment, Intimate partner violence, Victimization, Personality, Mental health, Causal inference, Mediation

## Abstract

**Background:**

Childhood maltreatment elevates risk of later intimate partner violence (IPV) victimization, both major psychiatric risk factors. Personality and mental health have been proposed as mediators, but these pathways may be confounded by genetic and environmental influences shared across maltreatment, IPV victimization, and intermediary phenotypes. Here, we examined how maltreatment confers additional IPV risk beyond shared etiological liabilities.

**Methods:**

We analyzed data from the UK's Twins Early Development Study (*N* = 11,342, aged 22) and genome wide association study summary statistics. We used structural equation modeling (SEM), twin modeling, and Linkage Disequilibrium Score Regression to estimate phenotypic and etiological associations between maltreatment, IPV victimization, and eighteen intermediary phenotypes. In a genotyped subsample (*n* = 8406), we used Mendelian Randomization-Direction of Causation (MR-DoC) to examine causal mediation beyond genetic and environmental confounding. Lived-experience input informed design and interpretation.

**Findings:**

Childhood maltreatment and IPV victimization showed a moderate phenotypic association (*β* = 0.23), small-to-moderate heritability (*h*^*2*^_*twin*_ = 17–31%; *h*^*2*^_*SNP*_ = 1–4%), and strong genetic correlations with each other (*r*_*g*_ = 0.52–0.82) and with most intermediary phenotypes (*r*_*g*_ = 0.16–0.84). Two latent factors capturing shared variance across all intermediary phenotypes partially mediated the effect of maltreatment on IPV victimization in SEM (total indirect effect ∑*ab* = 0.11, 48% mediated, direct effect *c’* = 0.12, *p*_*adj*_<0.001). In MR-DoC, which further adjusts for confounding, we also observed mediation, and no significant direct effect remained (∑*ab* = 0.09, 65% mediated, *c’* = 0.05, *p*_*adj*_ = 0.161). Three mediators were robust across models examining individual mediators (separately or simultaneously): low well-being, conduct problems, and aggression (*ab* = 0.01–0.05).

**Interpretation:**

Maltreatment elevates risk of IPV victimization through its impacts on personality and mental health development, beyond risk conferred by shared genetic and environmental liabilities. Preventing IPV victimization among young people exposed to maltreatment requires greater understanding of how these psychological impacts operate within social transactional contexts, and developing targeted strategies that address them.

**Funding:**

British Academy/10.13039/501100000275Leverhulme Trust.

**Research in context**

**Evidence before this study**

We conducted a systematic literature search through PubMed and citation searching up to June 2025 to identify quantitative studies investigating candidate mediators of the association between childhood maltreatment and subsequent IPV victimization. We used the search terms: (“child∗ maltreatment” [All Fields] OR “child∗ abuse” [All Fields] OR “child∗ neglect” [All Fields]) AND (“intimate partner violence” [All Fields] OR “domestic abuse” [All Fields] OR “domestic violence” [All Fields] OR “dating violence” [All Fields]) AND (“mediation” [All Fields] OR “mediator” [All Fields]). The search returned 138 records, of which 29 were eligible studies. The evidence reviewed suggested that several personality and mental health phenotypes, such as low self-control and depressive symptoms, may mediate the association between childhood maltreatment and later IPV victimization, with modest indirect effects. However, most studies examined one or a few mediators, often in small samples of undergraduate students from the United States of America. No studies adopted a genetically informed approach to account for genetic and environmental confounding.

**Added value of this study**

Using complementary phenotypic and genetically informed methods in a large, population-based cohort of young adult twins from the UK, we were able to disentangle different ways in which childhood maltreatment and later IPV risk are associated. Our findings demonstrate that both childhood maltreatment and later IPV victimization are shaped by a combination of genetic and environmental influences. Moreover, common genetic and environmental influences contribute to vulnerability to both victimization experiences and also to intermediary personality and mental health phenotypes. Even after accounting for these common etiological influences, childhood maltreatment still impacts personality and mental health development in ways that can heighten interpersonal risk, particularly through its influence on well-being, conduct problems, and aggression.

**Implications of all the available evidence**

The available evidence underscores that the risk of experiencing maltreatment in childhood and IPV victimization in young adulthood is not random: these adverse exposures are correlated with individual-level vulnerabilities that are partly genetic in origin. This implies that universal prevention strategies alone are unlikely to suffice if they do not account for individual differences in how individuals navigate their environments. Our findings also corroborate evidence that childhood maltreatment and IPV victimization are longitudinally associated. They add to this evidence by showing that this association reflects both shared etiological influences and the effects of childhood maltreatment on later IPV risk through its impact on personality and mental health development. These findings highlight the need for early, mechanistically informed IPV prevention programmes targeted at those exposed to childhood maltreatment, which consider how maltreatment increases interpersonal risk, particularly within intimate relationships. More broadly, our findings point to actionable targets to advance global priorities in IPV prevention, particularly among young people, and in improving adolescent health and wellbeing, reinforcing the need for adolescent services explicitly addressing relational functioning to mitigate interpersonal risks. Since adolescent relationships are developmentally formative, effective IPV prevention at this stage has the potential not only to reduce harm in the short term, but also to promote healthier relationships and improved health across the life course. Future research should extend our investigation to more diverse populations and facilitate its translation into practice and policy.

## Introduction

Childhood maltreatment, including abuse or neglect by a parent or caregiver, can disrupt personality development and lead to mental health problems.^1^^,^^2^ It also strongly increases—three to six times—the risk of later intimate partner violence (IPV) victimization.^3^^,^^4^ Several studies have therefore explored whether personality and mental health difficulties might act as intermediary phenotypes, mediating the association between childhood maltreatment (hereafter, maltreatment) and IPV victimization. These studies have typically found small indirect effects, for example, reporting that personality traits like impulsivity (e.g., indirect effect: *ab* = 0.06, *p* = 0.01^5^) and mental health difficulties like depressive symptoms (*ab* = 0.03–0.04, *p* < 0.05^6^), partially mediate this association. However, existing research has notable limitations: it has relied mostly on U.S. samples, examined a narrow set of mediators, often in isolation, and employed designs that do not support causal inference. Traditional cross-sectional designs cannot establish temporal precedence, leaving open the possibility that putative mediators are actually consequences of IPV victimization or confounders that independently predispose to both maltreatment and IPV victimization. Mendelian Randomization Direction of Causation (MR-DoC^7^) is a genetically informed approach that can help address these limitations and test causal hypotheses using cross-sectional data whilst minimizing risk for genetic and environmental confounding and reverse causation, representing a valuable approach in epidemiological research when experimental approaches would be unethical.

Genetically informed research has shown that maltreatment and IPV victimization are influenced by heritable traits^8^, ^9^, ^10^, ^11^ and that genetic and environmental risk factors contribute to vulnerability to both experiences, with maltreatment exerting a modest impact on IPV victimization beyond this shared etiology.^4^ Since the genetic makeup of one individual cannot directly influence the behavior of another, genetic influences on victimization reflect influences on phenotypes that correlate phenotypically and etiologically with victimization. This does not imply that genes predestine victimization or that individuals are in any way responsible for their victimization; rather, certain heritable traits, in interaction with environmental factors, may shape an individual's likelihood of encountering unsafe situations, and their responses to such situations. For example, genetic factors influence personality traits, which in turn influence relationship dynamics.^12^ Genetically informed research has identified specific phenotypes that may contribute to the heritable components of maltreatment and IPV victimization. Associations have been estimated between maltreatment and genetic risk for schizophrenia,^13^ autism spectrum disorder (ASD), post-traumatic stress disorder (PTSD),^14^ depression, and anxiety.^15^ Studies have shown that the genetic components of ASD, PTSD, risk tolerance, and well-being collectively account for 58% of the SNP-based heritability of maltreatment,^16^ and that 55–95% of genetic variants influencing maltreatment also influence ASD, depression, schizophrenia, borderline personality disorder, and attention-deficit/hyperactivity disorder (ADHD).^17^ Additionally, it has been shown that genetic factors contribute to the covariance between IPV victimization and depression,^11^ and that women in the highest quintile of genetic liability for depression, schizophrenia, ADHD, neuroticism, and overall mental illness also exhibited the highest likelihood of IPV victimization.^18^ While these shared genetic liabilities are not deterministic, nor do they imply immutability, they do not offer clear levers for intervention because the polygenic nature of risk makes it difficult to prioritise specific phenotypes. Identifying personality and mental health phenotypes that both reflect shared liabilities and are additionally shaped by maltreatment can more directly point to mechanisms that could be targeted in indicated preventive interventions. For example, if personality emerges as a significant mediator, this would suggest that personality-targeted interventions for young people exposed to maltreatment may be more effective than universal approaches in reducing IPV risk.

Altogether, phenotypic research has shown that personality and mental health phenotypes are candidate mediators of the pathway from maltreatment to IPV victimization, while genetically informed research has shown that maltreatment, IPV victimization, and intermediary phenotypes share genetic and environmental influences. The current study combined these research strands to:

1) Examine phenotypic and etiological associations between maltreatment, IPV victimization, and a wide range of personality and mental health phenotypes. We predicted that maltreatment and IPV victimization would show a moderate phenotypic association, as well as moderate heritability estimates and genetic correlations with each other and with intermediate phenotypes.
2) Identify phenotypes that mediate the association between maltreatment and IPV victimization beyond shared etiological influences. We aimed to replicate previous evidence of a modest effect of maltreatment on IPV victimization after accounting for their shared etiology^4^ while additionally addressing the gap in understanding the mechanisms through which this effect operates. As no study has simultaneously explored a comparably wide range of mediators, we did not make specific predictions. We did not examine sex differences in mediating effects since phenotypic and etiological associations between maltreatment and IPV victimization have been shown to be consistent across sexes.^3^^,^^4^

## Methods

### Participants

We used data from the age 21 wave of the Twins Early Development Study (TEDS), a cohort of twins born in England and Wales in 1994–1996. Participants were recruited through the Office for National Statistics. Response rates ranged from 84.40% at first contact to 75.60% of the original sample at age 21. Zygosity was determined using a parent-reported physical similarity questionnaire with 95% accuracy or DNA marker testing. See^19^^,^^20^ for recruitment procedures and sample representativeness,^21^ for genotyping procedures.

From the original sample with maltreatment and IPV victimization data (*N* = 11,770), we excluded 468 participants due to serious medical/perinatal conditions or missing zygosity. Sample size for phenotypic and twin analyses was *N* = 11,342 (5671 twin pairs), including 3976 monozygotic twins (63.98% women), 3640 same-sex dizygotic twins (62.31% women), 3726 different-sex dizygotic twins. Participants (Table 1) were 22 years old (*M* = 22.54, *SD* = 0.95). Most were white (93.77%), attending university (65.84%), and heterosexual (76.54% of women, 83.51% of men). Information on relationship history was obtained from a single item asking participants how many intimate relationships they had been in. Of the total sample, 19% (*n* = 2102) had missing data on this item; among respondents, 16% (*n* = 1441/9240) indicated they had never been in an intimate relationship. However, of these individuals, 82% (*n* = 1182) provided IPV victimization data, with 35% (*n* = 414) reporting at least one form of IPV. This suggests that IPV may have occurred within the context of dating relationships or brief encounters that participants did not classify as ‘intimate relationships’. This left 7% of our sample (*n* = 768) who reported both never having been in a relationship and no IPV victimization.

**Table 1:** Demographic information.

	n	%
Zygosity		
MZ female	2544	22.43%
MZ male	1432	12.63%
DZ female	2268	20.00%
DZ male	1372	12.10%
DZ different-sex	3726	32.85%
Missing	0	0.00%
Total study sample	11,342	
Ethnicity		
Non-White	704	6.23%
White	10,604	93.77%
Missing	34	0.30%
Total respondents	11,308	
Education		
No qualification	34	0.38%
GCSEs (any grades)	731	8.61%
A-levels or equivalent	1445	16.29%
Sub-degree higher education (CertHE, DipHE, HNC)	821	9.25%
First degree (e.g., BA or BSc)	4529	51.04%
Master's degree, PGCE or equivalent	1234	13.91%
PhD	80	0.90%
Missing	2468	27.81%
Total respondents	8874	
Sexual orientation		
Heterosexual	7276	79.75%
Gay/Lesbian	324	3.55%
Bisexual	1393	15.27%
Little or no sexual attraction	130	1.42%
Unsure or I do not know	70	0.77%
Missing	2219	24.32%
Total respondents	9123	
Relationship status		
Single	3830	41.65%
Dating non-exclusively	580	6.31%
In exclusive relationship	3299	35.88%
Living with partner	1361	14.80%
Married	111	1.21%
Widowed/separated/divorced	14	0.15%
Missing	2147	
N of respondents	9195	

*Note*. MZ, Monozygotic; DZ, Dizygotic; GCSEs, General Certificate of Secondary Education, qualifications typically taken at age 16; A-levels, qualification taken at age 18, required for university entry. HNC, Higher National Certificate and CertHE = Certificate of Higher Education, post-secondary qualifications; DipHE, Diploma of Higher Education; Foundation = degree equivalent to two years of undergraduate study. PGCE, Postgraduate Certificate in Education.

A subsample (*n* = 8406) who were genotyped and passed quality control were included in linkage disequilibrium score regression (LDSC), polygenic score (PGS) regression, and MR-DoC analyses.

### Ethics approval

TEDS was approved by King's College London's ethics committee (number PNM/09/10–104, 09/06/2021). Parents, teachers, and twins provided informed consent at different waves.

### Lived-experience involvement

Lived-experience experts were consulted through qualitative research (Supplementary Materials SM1).^22^ Their insights on the psychological factors mediating the association between maltreatment and IPV victimization informed the selection of mediators and interpretation of the current findings, alongside existing research.

### Measures

Choice of primary measures reflected our evidence review, lived-experience input, and availability in TEDS. Measures were selected by TEDS researchers for their validity, widespread use, accessibility, and suitability for large-scale research. We analyzed measures self-reported by twins (see Supplementary Table ST1 for items and references; ST2 for distribution of scores; ST3 for psychometric properties).

Maltreatment was assessed retrospectively using items adapted from the Avon Longitudinal Study of Parents and Children (ALSPAC) ‘Life at 22+’ questionnaire. Items assessed physical abuse (5 items; e.g., [when you were a child, how often] ‘Did an adult hit you so hard it left you with bruises or marks?’) and emotional abuse (3 items; e.g., ‘Did an adult say hurtful or insulting things to you?’) on a 5-point ordinal scale ranging from 0 (Never) to 4 (Very often). Twin concordance for maltreatment was moderate, with 23.96% of pairs showing discordance (1 standard deviation difference in exposure levels). Within-pair variation supports the validity of twin analyses, indicating that although maltreatment often arises within shared family contexts, twins can differ substantially in their experiences, suggesting contributions from both familial and individual-specific influences. IPV victimization by a current or past partner was measured using six items from the Centers for Disease Control and Prevention (CDC) Violence Prevention questionnaire. Items assessed physical abuse (1 item: ‘[Your partner, current or past] pushed, hit, kicked, or otherwise physically hurt you’), emotional abuse (2 items; e.g., ‘Sometimes said insulting things or threatened you’), and controlling behavior (3 items; e.g., ‘Tried to keep you away from your family or friends’) a 5-point ordinal scale ranging from 1 (Strongly disagree) to 5 (Strongly agree). Composite scores of maltreatment and IPV victimization were calculated as the mean of scale items (requiring ≥50% items non-missing) with higher scores indicating greater exposure.

Candidate mediators (ST1) were selected on theoretical and empirical grounds and comprised personality traits and mental health difficulties (e.g., elevated impulsivity or anxiety) that are both plausible consequences of maltreatment and potential contributors to relationship difficulties. We measured personality using the Big 5 Personality scale, assessing neuroticism, extraversion, openness, agreeableness, and conscientiousness. We measured self-control using a shortened version of the brief Self-Control Measure and the Consideration of Future Consequences Scale, and risk-taking using the Risk Taking Index. We measured anxiety, peer problems, hyperactivity, and conduct problems using the Strengths and Difficulties Questionnaire, depressive symptoms using the Short Mood and Feeling Questionnaire and aggression using the Brief Aggression Questionnaire. We measured antisocial behavior using the Edinburgh Study of Youth Transitions and Crime, ADHD using the Conners Hyperactivity scale, Inattention and Hyperactivity subscales and psychotic experiences using the Specific Psychotic Experiences Questionnaire, Paranoia and Hallucination subscales. We measured subjective well-being (hereafter, well-being) using three subscales from the Contentment with Life Assessment Scale: Love and Relationships, Community, and Financial Wellbeing.

For genetically informed mediation, we used a polygenic score (PGS) for maltreatment created based on publicly available genome wide association study (GWAS) summary statistics of maltreatment, which did not include TEDS in the discovery sample.^23^ Following quality control of summary statistics and matching with the target dataset, 1,162,498 single nucleotide polymorphisms (SNPs) were retained for PGS calculation.

### Statistical analyses

**Fig. 1:**
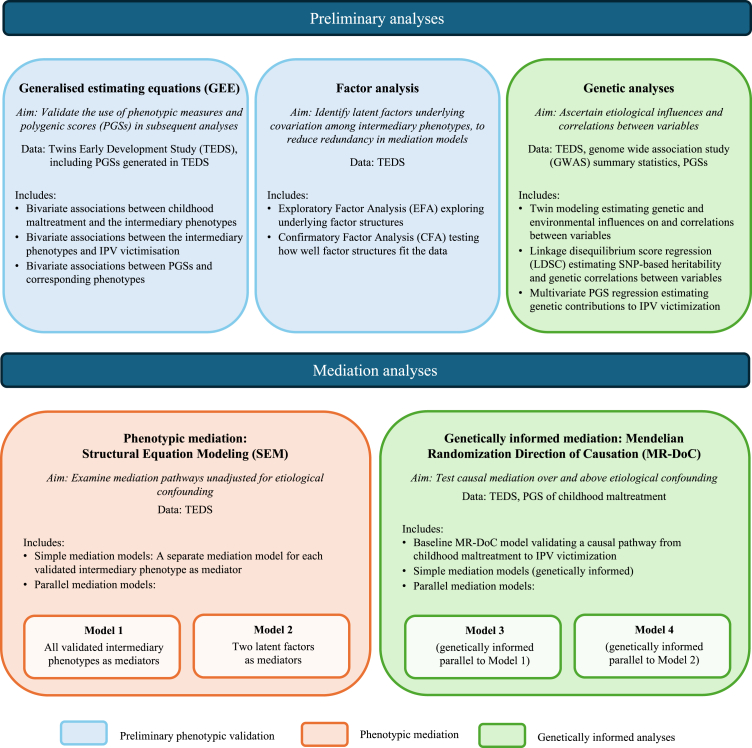
Overview of the statistical analyses.

An overview of the analyses is provided in Fig. 1.

#### Preliminary analyses

Following data preparation, including multiple imputation (Supplementary Materials SM2), we used GEE (SM3) and ordinary least square (OLS) regression to validate the use of phenotypic measures and PGSs in subsequent analyses (see ST4–ST6 for diagnostics). First, we estimated bivariate associations between maltreatment (specified as the predictor), each intermediary phenotype, and IPV victimization (specified individually as outcomes). We then estimated bivariate associations between each intermediary phenotype (as the predictor) and IPV victimization (as the outcome). To explore whether phenotypic associations differed by sexual orientation, we tested moderation using a binary indicator (0 = heterosexual, 1 = gay/lesbian or bisexual) and its interaction with the predictor. To assess whether modeling maltreatment continuously might obscure effects associated with relatively higher exposure, we compared associations between groups reporting higher (*n* = 5675) and lower (*n* = 5667) maltreatment levels, dichotomized using a median split to ensure balanced sample sizes and statistical power. In sensitivity analyses, we estimated phenotypic associations after excluding the 768 participants who reported never having been in a relationship and no IPV victimization. Finally, we estimated bivariate associations between each PGS and its corresponding variable within TEDS.

##### Factor analysis

We used exploratory and confirmatory factor analysis (SM4) to estimate latent factors underlying intermediary phenotypes, reducing redundancy in mediation models.

##### Genetic analyses

We used twin modeling in TEDS (SM5) and LDSC based on recent GWAS summary statistics (SM6) to estimate heritability and genetic correlations. We used multivariate PGS regression (SM7) to examine whether genetic variants associated with maltreatment and intermediary phenotypes also contributed to IPV victimization.

#### Mediation analyses

##### Structural equation modeling (SEM)

We used SEM to examine phenotypic mediation of the association between maltreatment and IPV victimization, unadjusted for confounding. This method was chosen to allow explicit modeling of measurement error in the mediator(s), a critical advantage given our self-report measures. It also accommodates the data structure of twins nested within families and extends naturally to the MR-DoC model, facilitating comparison between phenotypic and genetically informed results. We examined the effect of maltreatment on the mediator (*a*), the effect of the mediator on IPV victimization (*b*), the total effect of maltreatment on IPV victimization (*c*), its direct effect controlling for the mediator (*c′*) and its indirect effect, which captures the portion of the effect transmitted via the mediator (*ab*). We accounted for the non-independence of observations using a family identifier as the clustering variable.^24^ We fitted separate simple mediation models for each phenotype associated with maltreatment and IPV victimization in GEE. We also fitted two parallel mediation models. Model 1 simultaneously incorporated all significant mediators from simple mediation. In Model 2, all phenotypes were included and combined into two latent factors capturing their covariance (factor loadings in ST7), which were specified as mediators.

##### MR-DoC

We used MR-DoC^7^ (SM8, illustrated in Supplementary Figure SF1) to test whether mediation effects identified phenotypically remained significant after accounting for reverse causation and etiological influences shared with maltreatment and IPV victimization, such as shared environmental factors (e.g., neighborhood deprivation exerting an equivalent influence on both twins) elevating risk for both exposures. MR-DoC combines two quasi-experimental causal inference methods—Mendelian Randomization (MR^25^^,^^26^) and Direction of Causation (DoC^27^^,^^28^) twin modeling—to leverage the strengths and minimize biases inherent in each method.^7^^,^^29^ It is particularly useful for assessing mediation because it can help distinguish between three scenarios: i) the mediator is a consequence of both exposure and outcome (not a true mediator), ii) the mediator reflects shared genetic or environmental liability (confounding), or iii) the mediator causally transmits the effect of the exposure to the outcome (true mediation).

We first estimated a baseline MR-DoC model to validate the effect of maltreatment on IPV victimization beyond their shared etiology. Each significant mediator in phenotypic simple mediation was then examined in a separate MR-DoC simple mediation model. For mediators showing non-significant shared environmental influences in twin analyses (all but anxiety and peer problems), we constrained shared environmental components and correlations to zero to reduce model instability and prevent unreliable estimates (estimates from unconstrained models were consistent; see ST16). We fitted two MR-DoC parallel mediation models: Model 3 included all significant mediators in Model 1; Model 4 specified two latent factors as mediators, as in Model 2 (factor loadings in ST7).

We tested whether mediation effects in Models 2 and 4 differed by maltreatment levels by comparing two versions of each model, one with mediation paths freely estimated across higher and lower maltreatment groups and one with paths constrained equal, using likelihood ratio tests. MR-DoC analyses by sexual orientation were precluded due to the relatively small number of sexual minority participants (*n* = 1717).

Across mediation analyses, we applied Bonferroni correction, adopting a significance threshold of *p*_*adj*_ = 0.003 (0.05/18 candidate mediators).

Statistical analyses were conducted in R studio, version 4.4.2.^30^

### Role of the funding source

The funders had no role in study design, data collection, data analysis, interpretation, or writing.

## Results

### Preliminary analyses

Preliminary analyses suggested: i) significant phenotypic associations between maltreatment, IPV victimization, and our candidate mediators, as well as between PGSs and corresponding phenotypes ii) two latent factors underlying the phenotypic covariance between the candidate mediators, iii) small-to-moderate heritability estimates and strong genetic correlations between victimization experiences and candidate mediators, particularly psychotic experiences, externalizing behaviors, well-being and conscientiousness.

#### GEE

GEE (SM3) indicated a significant phenotypic association between maltreatment and IPV victimization (*β* = 0.23, 95% CIs [0.21, 0.25], which did not differ between individuals reporting higher vs. lower levels of maltreatment (above vs. below the median; *p* = 0.25), and between these variables and each intermediary phenotype except openness, which we thus excluded from mediation analyses. Maltreatment showed the strongest positive associations with psychotic experiences, ADHD, and depressive symptoms (*β* = 0.24–40), and the strongest negative associations with self-control, well-being, conscientiousness, and agreeableness (*β* = −0.19–0.13). IPV victimization showed the strongest positive associations with psychotic experiences and depressive symptoms, anxiety and peer problems, and conduct problems (*β* = 0.23–0.26), and the strongest negative associations with well-being, self-control, and conscientiousness (*β* = −21–0.12). The strength of association between maltreatment and IPV victimization did not differ significantly by sexual orientation (*p* = 0.808 for interaction term). Excluding the 768 participants who reported never having been in an intimate relationship and no IPV victimization yielded consistent results. GEE also confirmed associations between PGSs and corresponding phenotypes in TEDS (*β* = 0.05–0.15), except for conscientiousness and schizophrenia (*p*_*adj*_ > 0.01), which we thus excluded from PGS regression.

#### Factor analysis

We estimated two latent factors underlying the covariance between phenotypic measures (SM4): One, interpreted as negative/disordered affect, showed high loadings on anxiety, depressive symptoms, neuroticism, low extraversion, peer problems, psychotic experiences, and low well-being. The second factor, interpreted as externalizing tendencies, showed high loadings on risk-taking, low self-control, ADHD, conduct problems, low conscientiousness, aggression, hyperactivity, low consideration of future consequences, low agreeableness, antisocial behavior, and high openness.

#### Genetic analyses

Twin models (SM5) indicated small-to-moderate heritability estimates across variables (*h*^*2*^ = 0.17–0.47). Genetic, shared environmental, and nonshared environmental influences each accounted for about a third of the variance in maltreatment. Genetic and shared environmental influences were modest for IPV victimization (17%, 7%); nonshared environmental influences were large (76%). Maltreatment and IPV victimization showed strong genetic, moderate shared environmental, and small nonshared environmental correlations (*r*_*g*_ = 0.52 [0.52, 0.68], *r*_*c*_ = 0.42 [0.42, 0.74], *r*_*e*_ = 0.10 [0.07, 0.13]). Both showed the strongest positive genetic correlations with psychotic experiences and antisocial behavior (*r*_*g*_ = 0.44–0.64), and the strongest negative genetic correlations with well-being and conscientiousness (*r*_*g*_ = −0.39 to −0.36).

LDSC (SM6) estimated smaller heritability estimates (*h*^*2*^_*SNP*_ = 0.01–0.20) but a similar pattern of genetic correlations, with summary statistics for maltreatment and IPV victimization showing strong genetic correlations with summary statistics for externalizing behaviors (*r*_*g*_ = 0.42–0.61) and strong negative genetic correlations with well-being (*r*_*g*_ = -0.34 to −0.54). In PGS regression (SM7), genetic risk for externalizing behaviors, depressive symptoms, and ADHD predicted IPV victimization.

### Mediation analyses

Mediation analyses suggested: i) evidence for a potential causal effect of maltreatment on IPV victimization independent of their shared etiology, ii) substantial mediation of this effect via personality and mental health phenotypes, explaining 65% of the total effect; iii) robust mediation by low well-being, conduct problems, and aggression.

#### Phenotypic mediation (SEM)

Significant phenotypic mediation effects were found across simple mediation models (ST17). The total effect of maltreatment on IPV victimization was moderate (*c* = 0.24 [0.22, 0.25]), direct effects were small-to-moderate (*c'* = 0.16–0.23), indirect effects were small (*ab* = 0.01–0.08). Psychotic experiences, depressive symptoms, and conduct problems showed the strongest indirect effects (*ab* = 0.05–0.08).

**Fig. 2:**
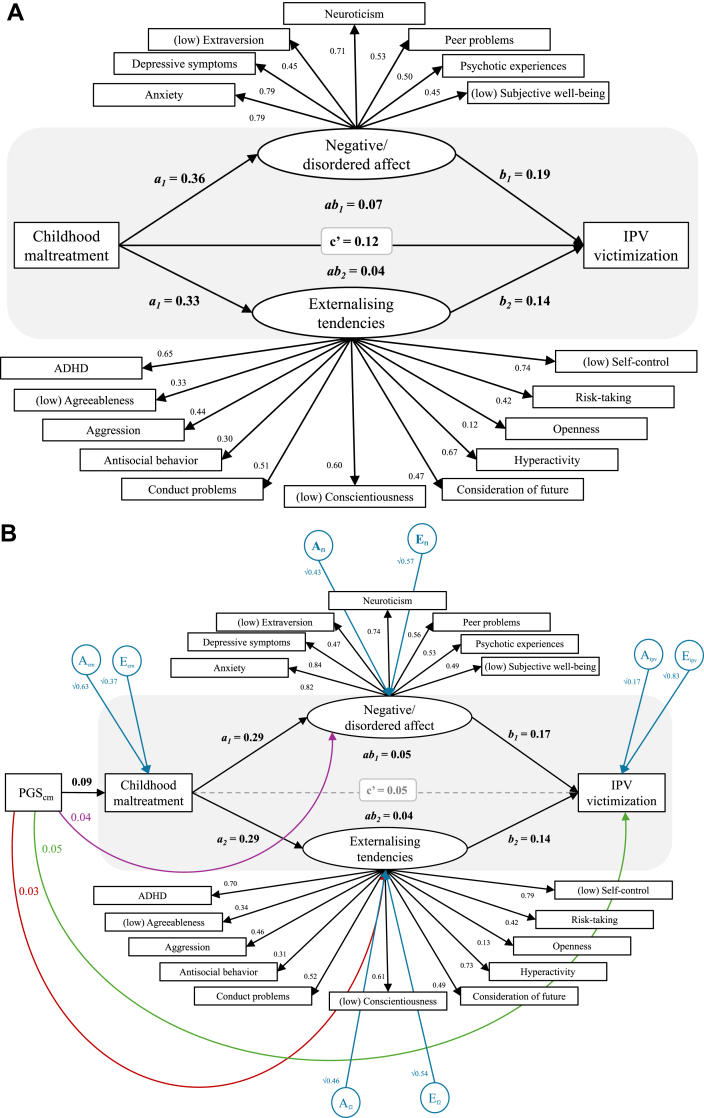
Panel a = Phenotypic parallel mediation (SEM) latent factors model (Model 2); Panel b = Genetically informed parallel mediation (MR-DoC) latent factors model (Model 4). In both panels: *a* = effect of maltreatment on the mediator; *b* = effect of the mediator on intimate partner violence (IPV) victimization, *c′* = direct effect of maltreatment on IPV victimization controlling for the mediator; *ab* = indirect effect, i.e. the portion of the effect of maltreatment on IPV victimization transmitted via the mediator. Abbreviations: IPV, Intimate partner violence; ADHD, Attention-Deficit/Hyperactivity Disorder; Consideration of future, Consideration of future consequences, a measure of self-control; PGS, polygenic score. Suffixes denote estimates relative to: ‘cm’ = childhood maltreatment, ‘ipv’ = intimate partner violence victimization, ‘f1’ = Factor 1 (negative/disordered affect), ‘f2’ = Factor 2 (externalizing tendencies). A = additive genetic variance component; E = non-shared environmental variance component. AE paths are presented as standardized path coefficients; when squared, they represent the proportion of variance explained. Rectangles represent observed variables; ovals represent latent variables. Dashed lines denote non-significant paths (*p*_*adj*_ > 0.003). For confidence intervals around the estimates, see ST7 (factor loadings), ST18 (Model 2), and ST19 (Model 4).

Across parallel mediation models (ST18), the total effect of maltreatment on IPV victimization was moderate (Model 1: *c* = 0.24 [0.21, 0.26]; Model 2: *c* = 0.24 [0.22, 0.25]), its direct effect was small (Model 1: *c'* = 0.10 [0.07, 0.12]; Model 2: *c'* = 0.12 [0.11, 0.14]). In Model 1, the total indirect effect was modest (∑*ab* = 0.14 [0.12, 0.15], 58% mediated). Psychotic experiences, peer problems, well-being, aggression, conduct problems, and risk-taking showed significant indirect effects (*ab* = 0.01–0.03). In Model 2 (Fig. 2a), the total indirect effect was small (∑*ab* = 0.11 [0.10, 0.12], 48% mediated), as was the direct effect (*c'* = 0.12 [0.11, 0.15]). Both latent factors showed significant indirect effects (*ab* = 0.04–0.07). We found no significant differences in mediation effects between higher vs. lower maltreatment groups (*p* = 0.012, exceeding our adjusted significance threshold of *p*_*adj*_ = 0.003).

#### Genetically informed mediation (MR-DoC)

The baseline MR-DoC model supported a small effect of maltreatment on IPV victimization independent of their shared etiology (0.14 [0.10, 0.18]). We found a small instrumental path from the PGS for maltreatment to maltreatment (0.09 [0.07, 0.11]) and a small pleiotropic path from the PGS for maltreatment to IPV victimization (0.06 [0.04, 0.07]).

Across MR-DoC simple mediation models (Table 2), the total effect of maltreatment on IPV victimization was small (*c* = 0.14 [0.13, 0.15]) as was its direct effect (*c’* = 0.09–0.14). Indirect effects were small (0.02–0.06), with the strongest indirect effects for psychotic experiences, well-being, and depressive symptoms (0.04–0.06). All paths were significant except *a* paths from maltreatment to extraversion and consideration of future consequences, *b* paths from extraversion, agreeableness, and antisocial behavior to IPV victimization, and the *c’* path via psychotic experiences.

**Table 2:** Estimates, genetically informed simple mediation (MR-DoC) models.

Mediator	*a*	lCI	uCI	*b*	lCI	uCI	*c'*	lCI	uCI	*ab*	lCI	uCI	Pleiotropic paths	lCI	uCI
Neuroticism	**0.19**	**0.15**	**0.22**	**0.12**	**0.10**	**0.15**	**0.12**	**0.08**	**0.16**	**0.02**	**0.02**	**0.03**	**0.04**	**0.02**	**0.05**
Extraversion	−0.07	−0.10	−0.03	−0.02	−0.04	0.01	**0.14**	**0.10**	**0.18**	0.00	0.00	0.00	−0.03	−0.04	−0.01
Agreeableness	**−0.15**	**−0.19**	**−0.11**	−0.03	−0.05	0.00	**0.14**	**0.10**	**0.18**	0.00	0.00	0.01	−0.01	−0.02	0.01
Conscientiousness	**−0.12**	**−0.16**	**−0.08**	**−0.06**	**−0.09**	**−0.04**	**0.14**	**0.10**	**0.18**	**0.01**	**0.01**	**0.01**	−0.02	−0.04	−0.01
Self-control	**−0.19**	**−0.23**	**−0.15**	**−0.12**	**−0.15**	**−0.09**	**0.12**	**0.08**	**0.16**	**0.02**	**0.02**	**0.03**	−0.02	−0.03	0.00
Consideration of future consequences	−0.06	−0.10	−0.02	**−0.07**	**−0.09**	**−0.05**	**0.14**	**0.10**	**0.18**	0.00	0.00	0.01	0.00	−0.01	0.02
Risk-taking	**0.16**	**0.12**	**0.19**	**0.10**	**0.07**	**0.13**	**0.13**	**0.09**	**0.17**	**0.02**	**0.01**	**0.02**	0.00	−0.02	0.01
Anxiety	**0.19**	**0.15**	**0.23**	**0.13**	**0.10**	**0.16**	**0.12**	**0.08**	**0.15**	**0.02**	**0.02**	**0.03**	0.03	0.01	0.04
Peer problems	**0.14**	**0.11**	**0.18**	**0.12**	**0.09**	**0.15**	**0.13**	**0.09**	**0.16**	**0.02**	**0.01**	**0.02**	0.05	0.03	0.07
Hyperactivity	**0.16**	**0.12**	**0.20**	**0.10**	**0.08**	**0.13**	**0.13**	**0.09**	**0.17**	**0.02**	**0.01**	**0.02**	0.03	0.02	0.05
Conduct problems	**0.24**	**0.21**	**0.28**	**0.12**	**0.09**	**0.14**	**0.11**	**0.08**	**0.15**	**0.03**	**0.02**	**0.03**	0.02	0.01	0.04
Depressive symptoms	**0.27**	**0.23**	**0.31**	**0.13**	**0.10**	**0.15**	**0.11**	**0.07**	**0.15**	**0.04**	**0.03**	**0.04**	0.03	0.01	0.04
Aggression	**0.15**	**0.12**	**0.19**	**0.09**	**0.07**	**0.12**	**0.13**	**0.09**	**0.17**	**0.01**	**0.01**	**0.02**	**0.05**	**0.03**	**0.06**
Antisocial behavior	**0.21**	**0.17**	**0.25**	0.04	0.02	0.07	**0.14**	**0.10**	**0.18**	**0.01**	**0.01**	**0.01**	0.03	0.01	0.04
Attention-deficit hyperactivity disorder	**0.27**	**0.23**	**0.30**	**0.10**	**0.07**	**0.12**	**0.12**	**0.08**	**0.16**	**0.03**	**0.02**	**0.03**	0.03	0.01	0.04
Psychotic experiences	**0.46**	**0.43**	**0.50**	**0.13**	**0.10**	**0.15**	0.09	0.04	0.13	**0.06**	**0.05**	**0.07**	0.01	−0.01	0.02
Subjective well-being	**−0.22**	**−0.25**	**−0.18**	**−0.17**	**−0.20**	**−0.15**	**0.11**	**0.07**	**0.15**	**0.04**	**0.03**	**0.04**	0.00	−0.01	0.02

Note. Standardized estimates. a = effect of childhood maltreatment on the candidate mediator; b = effect of the candidate mediator on intimate partner violence (IPV) victimization; c’ = direct effect of maltreatment on IPV victimization controlling for the mediator; ab = indirect effect of maltreatment on IPV victimization via the mediator; lCI = 95% confidence intervals, lower bound; uCI = 95% confidence intervals, upper bound. In each model, the instrumental path from the PGS of childhood maltreatment to childhood maltreatment measured in TEDS was 0.09 (0.07, 0.11), the pleiotropic path from the PGS of childhood maltreatment to IPV victimization was 0.06 (0.04, 0.07). The total effect of maltreatment on IPV victimization was c = 0.14 (0.13, 0.15). Statistically significant estimates (padj <0.003) are highlighted in bold font.

We tested two MR-DoC parallel mediation models (Table 3). Model 3 included the six phenotypes that significantly mediated the pathway from maltreatment to IPV victimization in Model 1. Three of these—well-being, conduct problems, and aggression—showed significant indirect effects (0.02–0.03). The total effect of maltreatment on IPV victimization was small (*c* = 0.13 [0.10, 0.17]), as was the total indirect effect (∑*ab* = 0.10 [0.08, 0.11], 72% mediated). The direct effect was not significant (*c’* = 0.04 [0.00, 0.07], *p*_*ad*j_ = 1.000). In Model 4 (Fig. 2b), the total effect was small (*c* = 0.10 [0.14, 0.17]), as was the total indirect effect (∑*ab* = 0.09 [0.07, 0.10], 65% mediated). The direct effect was not significant (*c'* = 0.05 [0.01, 0.09], *p*_*adj*_ = 0.161). Both latent factors showed significant indirect effects (*ab* = 0.04–0.05). Model 4 differed by maltreatment level (*p* < 0.001), driven by a stronger effect of maltreatment on externalizing tendencies in the lower vs. higher maltreatment group (*b* = 0.34 [0.31, 0.37] vs. 0.23 [0.20, 0.26]).

**Table 3:** Estimates, genetically informed parallel mediation (MR-DoC) models (Models 3 and 4).

Mediator	*a*	lCI	uCI	*b*	lCI	uCI	*ab*	lCI	uCI	Pleiotropic path	lCI	uCI
**Model 3**												
Risk-taking	**0.16**	**0.13**	**0.19**	0.04	0.01	0.07	0.01	0.00	0.01	0.00	−0.02	0.02
Peer problems	**0.11**	**0.07**	**0.14**	0.05	0.03	0.08	0.01	0.00	0.01	**0.05**	**0.04**	**0.07**
Conduct problems	**0.21**	**0.17**	**0.24**	**0.08**	**0.06**	**0.10**	**0.03**	**0.02**	**0.04**	0.03	0.01	0.04
Aggression	**0.16**	**0.13**	**0.20**	0.04	0.01	0.06	**0.02**	**0.01**	**0.02**	**0.05**	**0.03**	**0.06**
Psychotic experiences	**0.47**	**0.44**	**0.50**	**0.07**	**0.05**	**0.10**	0.01	0.00	0.01	0.01	−0.01	0.02
Subjective well-being	**−0.21**	**−0.24**	**−0.18**	**−0.13**	**−0.16**	**−0.11**	**0.03**	**0.02**	**0.05**	0.00	−0.01	0.02
**Model 4**												
Negative/disordered affect	**0.29**	**0.26**	**0.32**	**0.17**	**0.13**	**0.20**	**0.05**	**0.03**	**0.07**	**0.04**	**0.02**	**0.06**
Externalizing tendencies	**0.29**	**0.26**	**0.32**	**0.14**	**0.10**	**0.17**	**0.04**	**0.02**	**0.06**	**0.03**	**0.02**	**0.05**

*Note*. Standardized estimates. *a* = effect of childhood maltreatment on the candidate mediator; *b* = effect of the candidate mediator on intimate partner violence (IPV) victimization; *c’* = direct effect of maltreatment on IPV victimization controlling for the mediator; *ab* = indirect effect of maltreatment on IPV victimization via the mediator; lCI = 95% confidence intervals, lower bound; uCI = 95% confidence intervals, upper bound. Model 3 considered all intermediary phenotypes; Model 4 additionally estimated two latent factors accounting for their covariation. In Model 3, the total effect of maltreatment on IPV victimization was *c* = 0.13 (0.10, 0.17), the total indirect effect was ∑*ab* = 0.10 (0.08, 0.11; 72% mediated), and the direct effect controlling for all mediators was *c’* = 0.04 [0.00, 0.07], *p*_*ad*j_ = 1.000. In Model 4, the total effect was *c* = 0.10 (0.14, 0.17), the total indirect effect was ∑*ab* = 0.09 (0.07, 0.10; 65% mediated), and the direct effect controlling for the latent factors was *c'* = 0.05 (0.01, 0.09; *p* = 0.161). In both models, the instrumental path from the PGS of childhood maltreatment to childhood maltreatment measured in TEDS was 0.09 (0.08, 0.11) and the pleiotropic path from the PGS of childhood maltreatment to IPV victimization was 0.05 (0.04, 0.07). Statistically significant estimates (*p*_*adj*_ < 0.003) are highlighted in bold font.

Across MR-DoC models (model fit indices in ST19), the instrumental path and the pleiotropic path from the PGS for maltreatment to IPV victimization were consistent with the baseline MR-DoC model; pleiotropic paths to the mediators ranged between −0.03 (extraversion) and 0.05 (peer problems, aggression).

## Discussion

This study marks a significant advance in elucidating the processes linking maltreatment to later IPV victimization. In a large population-based twin sample, we examined how much of this association is attributable to common genetic and environmental liabilities. We explored a range of personality and mental health phenotypes as potential intermediaries, assessing the extent to which they reflect shared etiological risk or mediating mechanisms. Consistent with our first prediction, maltreatment and IPV victimization showed a moderate phenotypic association, moderate heritability, and substantial genetic overlap with each other and most intermediary phenotypes. Consistent with our second prediction, maltreatment had a modest impact on IPV victimization beyond shared etiological influences. However, no statistically significant direct effect remained once both shared etiological influences and intermediary phenotypes were accounted for. Intermediary phenotypes accounted for 65% of the association between maltreatment and IPV victimization. Although additional mediating mechanisms may exist beyond those examined here, this finding suggests that a substantial proportion of the effect of maltreatment operates through psychological pathways. Mediated effects were consistent across participants reporting lower vs. higher levels of maltreatment, meaning these effects were not solely driven by individuals with extreme exposure, but reflected a dimensional relationship across the sample.

Of the eighteen candidate mediators, three were statistically robust: low well-being, conduct problems, and aggression. Each of these traits showed phenotypic and genetic links to both maltreatment and IPV victimization, and significantly mediated the association between them. Low subjective well-being (dissatisfaction with one's relationships, community, and finances) is a common outcome of maltreatment and likely reflects impaired coping strategies needed to build and maintain an adaptive social architecture.^31^ Conduct problems and aggression—encompassing anger, hostility, and verbal and physical aggression—have been associated with early adversity and may reflect maladaptive responses to threatening or inconsistent caregiving environments, particularly among individuals with a predisposition to externalizing traits.^32^ Over time, such difficulties in emotional and behavioral regulation may increase vulnerability to further trauma, including IPV.^33^ Moreover, individuals displaying conduct problems or aggressive traits face increased risk of both IPV victimization and perpetration, including mutual violence within the same relationship, partly due to assortative partner selection.^34^ Our findings underscore the value of interventions targeting such traits. For example, parenting programs aimed at reducing childhood conduct problems have shown promise in reducing the risk of adolescent IPV involvement.^35^

Another noteworthy finding is that risk-taking, while a significant mediator in most models, did not retain significance in genetically informed parallel mediation. Research has frequently emphasized risk-taking—especially sexual risk-taking—as a proximal mechanism underlying revictimization.^36^ Our findings caution against attributing unique explanatory weight to risk-taking, which may inadvertently contribute to victim-blaming narratives which suggest that individuals are in some way responsible for their victimization. Lived-experience individuals report that risk-taking may reflect a desire to fulfill unmet emotional needs rather than a deliberate courting of danger.^22^ Our findings highlight that this coping strategy is strongly influenced by underlying dispositions, themselves shaped by genetic and environmental factors beyond individual control. Recognizing these influences may help shift narratives away from victim blaming, towards a more nuanced understanding of interpersonal risk that acknowledges the interaction between personal, structural, and contextual risk factors. Similarly, peer problems—social rejection, isolation, and conflict—did not retain significance in genetically informed parallel mediation, and may reflect manifestations of latent vulnerabilities operating across development. For example, maltreatment is more prevalent in socially deprived areas, where limited community resources can constrain opportunities to form supportive peer relationships,^37^ and this reduced social capital can increase risk of abusive intimate relationships.^38^

From a translational perspective, it is critical to recognize that genetic influences on maltreatment, IPV victimization, and intermediary traits do not mean these outcomes are predetermined. Rather, they heighten risk indirectly, alongside environmental factors, by shaping traits associated with interpersonal vulnerability. This underscores the need to consider individual differences when designing prevention strategies. Primary IPV prevention largely relies on universal school-based relationship education focused on general skills.^39^ However, one-size-fits all approaches are unlikely to meet the needs of at-risk individuals. Mechanistically informed targeted interventions addressing personality and mental health difficulties could be more effective, especially for young people with maltreatment histories. For example, programmes targeting well-being, conduct problems, and aggression may help disrupt interpersonal stress generation processes through which maltreatment elevates interpersonal risk.

### Strengths and limitations

Our large, genetically informed design indicates that the psychological pathways linking maltreatment to later IPV are best conceptualized as complex, involving genetic and environmental predispositions alongside personality and mental health vulnerabilities exacerbated by maltreatment. While the MR-DoC approach provides the most rigorous test of causality in these pathways to date—disentangling mediation from spurious associations arising from shared liabilities—caution in interpreting the findings is warranted. Although we implemented strategies to mitigate measurement error (e.g., using a PGS as an unconfounded instrument for maltreatment, and latent modeling of mediators), our analyses relied on cross-sectional self-report data. Replication in independent samples is needed, as are GWASs of maltreatment and IPV at different developmental stages. For example, we could not test reverse causation due to the absence of published GWAS summary statistics for IPV victimization—though a previous study using DoC twin modeling supports a directional effect from maltreatment to IPV victimization rather than the reverse.^4^ Given that experimental studies on this question are precluded on ethical grounds, quasi-experimental approaches like MR-DoC represent a statistically rigorous strategy for investigating causality.

Although comprehensive, our study did not examine specific cognitive mechanisms affected by maltreatment, such as reward sensitivity. However, as these mechanisms are closely related to the phenotypes examined, overlapping variance may have been captured. The reliance on validated but necessarily brief measures also limited depth of assessment, and the demographic composition of the sample, primarily heterosexual of white ancestry, may limit the generalizability of our findings. Since minorities may experience unique contextual risks, further research investigating specific mechanisms through which intermediary phenotypes operate in more diverse samples is warranted.^40^ Lastly, we did not assess IPV perpetration or partner characteristics, which constrains our understanding of partner violence in this sample. Prior research indicates that maltreatment increases risk for both IPV victimization and perpetration, with substantial overlap between the two.^41^ It is plausible that some of the vulnerabilities identified here may also be relevant for perpetration, but this requires a dedicated investigation.

### Conclusion

To our knowledge, this is the first study to combine comprehensive assessment of candidate mediators spanning personality and mental health domains with genetically informed causal inference methods, to investigate the psychological pathways underlying the association between maltreatment and later IPV victimization. We demonstrated that this association is accounted for by both etiological liabilities shared between victimization experiences, personality, and mental health, and the impact of maltreatment on psychological development beyond shared etiological liabilities. We showed that 65% of the total effect of maltreatment on IPV victimization operates via the measured psychological pathways. Findings offer a compelling foundation for developing indicated interventions for young people exposed to maltreatment aimed at preventing IPV through approaches that account for individual differences in childhood experiences, personality, and mental health.

## Contributors

Conceptualization: Pezzoli, Viding, Pingault, Barkhuizen, Oginni; Funding acquisition: Pezzoli, Viding, Pingault; Project administration: Pezzoli; Methodology: Pezzoli, Barkhuizen, Oginni, Pingault, Viding; Data curation: Pezzoli; Formal analysis: Pezzoli, Barkhuizen; Writing–Original draft: Pezzoli; Visualization: Pezzoli; Writing–Review & editing: Pezzoli, Viding, McCrory, Oginni, Barkhuizen, Pingault.

## Data sharing statement

This study is part of a larger project pre-registered on the Open Science Framework, at osf.io/byqm4. The TEDS data are available upon request at teds.ac.uk/researchers/teds-data-access-policy. The code for this study is available at osf.io/4rv3x.

## Use of generative AI

During the preparation of this work, the authors used OpenAI's ChatGPT (GPT-4, June 2024) to assist with improving readability. After using this tool, the authors reviewed and edited the content as needed and take full responsibility for the content of the publication.

## Declaration of interests

None.
