# Supplementary material for: Personality and mental health as mediators linking childhood maltreatment to intimate partner violence victimization: a Mendelian randomization–direction of causation twin study
Source: Lancet Reg Health Eur. 2026 Mar 26;66:101653. doi: 10.1016/j.lanepe.2026.101653 (PMC13185925; doi:10.1016/j.lanepe.2026.101653)
Supplement: Supplementary Materials [file mmc1.docx]

**The role of personality and mental health in the association between childhood maltreatment and later intimate partner violence victimization**

**Supplementary Materials**

Patrizia Pezzoli*, Wikus Barkhuizen, Olakunle Oginni, Jean-Baptiste Pingault, Eamon McCrory, Essi Viding

*Corresponding author: p.pezzoli@ucl.ac.uk

Table of Contents

[Supplementary Table 1 (ST1). Questionnaire measures 4](#_Toc212821838)

[Supplementary Materials 1. Lived-experience contribution 12](#_Toc212821839)

[Supplementary Materials 2. Data preparation 12](#_Toc212821840)

[Supplementary Table 2 (ST2). Distribution of raw phenotypic composite scores and polygenic scores 14](#_Toc212821841)

[Supplementary Table 3 (ST3). Psychometric properties of raw composite scores 16](#_Toc212821842)

[Supplementary Materials 3. Generalized Estimating Equations 18](#_Toc212821843)

[Methods 18](#_Toc212821844)

[Results 18](#_Toc212821845)

[Supplementary Table 4 (ST4). Generalized Estimating Equations results, phenotypic associations with maltreatment 20](#_Toc212821846)

[Supplementary Table 5 (ST5). Generalized Estimating Equations results, phenotypic associations with intimate partner violence victimization 21](#_Toc212821847)

[Supplementary Table 6 (ST6). Polygenic score instrument diagnostics 22](#_Toc212821848)

[Supplementary Materials 4. Factor analysis 24](#_Toc212821849)

[Methods 24](#_Toc212821850)

[Results 24](#_Toc212821851)

[Supplementary Table 7 (ST7). Factor loadings, phenotypic and genetically informed latent factor models 26](#_Toc212821852)

[Supplementary Table 8 (ST8). Model fit indices, confirmatory factor analyses 27](#_Toc212821853)

[Supplementary Materials 5. Twin modeling 28](#_Toc212821854)

[Methods 28](#_Toc212821855)

[Results 28](#_Toc212821856)

[Supplementary Table 9 (ST9). Estimates, univariate twin models 29](#_Toc212821857)

[Supplementary Table 10 (ST10). Model fit indices, univariate twin models 30](#_Toc212821858)

[Supplementary Table 11 (ST11). Variance components and etiological correlations, separate multivariate twin models 32](#_Toc212821859)

[Supplementary Table 12 (ST12). Model fit indices, multivariate twin models 34](#_Toc212821860)

[Supplementary Materials 6. Linkage Disequilibrium score regression (LDSC) 36](#_Toc212821861)

[Methods 36](#_Toc212821862)

[Results 37](#_Toc212821863)

[Supplementary Table 13 (ST13). SNP heritability estimates, Linkage Disequilibrium score regression 38](#_Toc212821864)

[Supplementary Table 14 (ST14). Genetic correlations, Linkage Disequilibrium score regression 39](#_Toc212821865)

[Supplementary Materials 7. Polygenic score regression 40](#_Toc212821866)

[Methods 40](#_Toc212821867)

[Results 40](#_Toc212821868)

[Supplementary Table 15 (ST15). Estimates, polygenic score regression of intimate partner violence victimization on polygenic scores 41](#_Toc212821869)

[Supplementary Materials 8. Mendelian Randomization Direction of Causation 42](#_Toc212821870)

[Methods 42](#_Toc212821871)

[Supplementary Figure SF1. 44](#_Toc212821872)

[Results 46](#_Toc212821873)

[Supplementary Table S16 (ST16). Estimates, genetically informed simple mediation (MR-DoC) models with freely estimated shared environmental parameters 46](#_Toc212821874)

[Supplementary Table 17 (ST17). Estimates, phenotypic simple mediation (SEM) models 48](#_Toc212821875)

[Supplementary Table 18 (ST18). Estimates, phenotypic parallel mediation (SEM) models (Models 1 and 2) 49](#_Toc212821876)

[Supplementary Table 19 (ST19). Model fit indices, MR-DoC models 51](#_Toc212821877)

[References 53](#_Toc212821878)

## Supplementary Table 1 (ST1). Questionnaire measures

|  | Scale | Subscale | Item |
| --- | --- | --- | --- |
| Victimization experiences | | |  |
|  | Childhood maltreatment | |  |
|  | Life at 22+ questionnaire ^1^ | | When you were a child, how often... Response options: Never (0), Rarely (1), Sometimes (2), Often (3), Very often (4) |
|  |  |  | 1. Did an adult in your family shout at you? |
|  |  |  | 2. Did an adult say hurtful or insulting things to you? |
|  |  |  | 3. Did an adult push, grab or shove you? |
|  |  |  | 4. Did an adult smack you for discipline? |
|  |  |  | 5. Did an adult punish you in a way that seemed cruel? |
|  |  |  | 6. Did an adult threaten to kick, punch, or hit you with something that could hurt you, or physically attack you in another way? |
|  |  |  | 7. Did an adult actually kick, punch, or hit you with something that could hurt you, or physically attack you in another way? |
|  |  |  | 8. Did an adult hit you so hard it left you with bruises or marks? |
|  | Intimate partner violence victimization | |  |
|  | CDC Violence Prevention questionnaire ^2^ | | Your partner (current or past)... Response options: Strongly disagree (1), Disagree (2), Neither agree nor disagree (3), Agree (4), Strongly agree (5) |
|  |  |  | 1. Got very jealous or tried to control your life |
|  |  |  | 2. Tried to keep you away from your family or friends |
|  |  |  | 3. Sometimes said insulting things or threatened you |
|  |  |  | 4. Pushed, hit, kicked, or otherwise physically hurt you |
|  |  |  | 5. You were afraid to disagree with your partner (current or past) because you thought they might hurt you or other family members |
|  |  |  | 6. Made you feel scared or frightened |
| Personality | | |  |
|  | Big 5 Personality Scale ^3^ | | In terms of the following personality traits, please rate where you fall on the scale from low (1) to high (5) |
|  |  | Neuroticism | 1. Relaxed, unconcerned, cool (low) to fearful, apprehensive (high) |
|  |  |  | 2. Even-tempered (low) to angry, bitter |
|  |  |  | 3. Optimistic (low), pessimistic, glum (high) |
|  |  |  | 4. Self-assured, glib, shameless (low) to timid, embarrassed (high) |
|  |  |  | 5. Controlled, restrained (low) to tempted, urgency (high) |
|  |  |  | 6. Clear-thinking, fearless, unflappable (low) to helpless, fragile (high) |
|  |  | Extraversion | 1. Cool, aloof, indifferent (low) to cordial, affectionate, attached (high) |
|  |  |  | 2. Withdrawn, isolated (low) to sociable, outgoing (high) |
|  |  |  | 3. Unassuming, quiet, resigned (low) to dominant, forceful (high) |
|  |  |  | 4. Passive, lethargic (low) to vigorous, energetic, active (high) |
|  |  |  | 5. Cautious, monotonous, dull (low) to reckless, daring (high) |
|  |  |  | 6. Placid, anhedonic (low) to high-spirited (high) |
|  |  | Openness | 1. Practical, concrete (low) to dreamer, unrealistic, imaginative (high) |
|  |  |  | 2. Uninvolved, no artistic interest (low) to curious, appreciate art (high) |
|  |  |  | 3. Constricted, unaware, alexytymic (low) to self-aware (high) |
|  |  |  | 4. Routine, predictable, habitual, stubborn (low) to unconventional, eccentric (high) |
|  |  |  | 5. Pragmatic, rigid (low) to strange, odd, peculiar, creative (high) |
|  |  |  | 6. Traditional, inflexible, dogmatic, rigid (low) to permissive, broad-minded (high) |
|  |  | Agreeableness | 1. Sceptical, cynical, suspicious, paranoid (low) to gullible, naive, trusting (high) |
|  |  |  | 2. Cunning, manipulative, deceptive (low) to confiding, honest (high) |
|  |  |  | 3. Stingy, selfish, greedy, exploitative (low) to sacrificial, giving (high) |
|  |  |  | 4. Oppositional, combative, aggressive (low) to docile, cooperative (high) |
|  |  |  | 5. Confident, boastful, arrogant (low) to meek, self-effacing, humble (high) |
|  |  |  | 6. Tough, callous, ruthless (low) to soft, empathetic (high) |
|  |  | Conscientiousness | 1. Lax, negligent (low) to perfectionist, efficient (high) |
|  |  |  | 2. Haphazard, disorganised, sloppy (low) to ordered, methodical, organised (high) |
|  |  |  | 3. Casual, undependable, unethical (low) to rigid, reliable, dependable (high) |
|  |  |  | 4. Aimless, desultory (low) to workaholic, ambitious (high) |
|  |  |  | 5. Hedonic, negligent (low) to dogged, devoted (high) |
|  |  |  | 6. Hasty, careless, rash (low) to cautious, ruminative, reflective (high) |
|  | Brief Self-Control Measure ^4^ | | Please rate the extent to which you match the following statements about your personality. Response options: Not at all (0), Slightly (1), Moderately (2), Quite (3), Very much (4) |
|  |  |  | I am good at resisting temptation |
|  |  |  | I have a hard time breaking bad habits |
|  |  |  | I am lazy |
|  |  |  | I say inappropriate things |
|  |  |  | Pleasure and fun sometimes keep me from getting work done |
|  |  |  | I have trouble concentrating |
|  | Consideration of Future Consequences Scale ^5^ | | Please rate the extent to which the following statements are characteristic of your personality from extremely uncharacteristic of me (1) to extremely characteristic of me (5) |
|  |  |  | 1.  I only act to satisfy immediate concerns, figuring the future will take care of itself |
|  |  |  | 2. I generally ignore warnings about possible future problems because I think the problems will be resolved before they reach crisis level |
|  |  |  | 3. I think that sacrificing now is usually unnecessary since future outcomes can be dealt with at a later time |
|  |  |  | 4. I only act to satisfy immediate concerns, figuring that I will take care of future problems that may occur at a later date |
|  | Risk-taking Index ^6^ | | How often do you take… Response options: Never (0), Rarely (2), Sometimes (3), Often (4), Very often (5) |
|  |  |  | 1. Recreational risks (e.g. skiing, skateboarding, skydiving, cliff-jumping/tombstoning)? |
|  |  |  | 2. Health risks (e.g. smoking, poor diet, high alcohol consumption, binge-drinking)? |
|  |  |  | 3. Career risks (e.g. quitting a job without another to go to)? |
|  |  |  | 4. Financial risks (e.g. gambling, risky investments)? |
|  |  |  | 5. Safety risks (e.g. fast driving, cycling without a helmet, being in a car without a seat belt)? |
|  |  |  | 6. Social risks (e.g. standing for election, publicly challenging a rule or decision)? |
| Mental health | | |  |
|  | Strengths and Difficulties Questionnaire ^7^ | | Please rate how true the following statements have been about you in the last six months. Response options: Not true (0), Quite true (1), Very true (2) |
|  |  | Emotional symptoms (Anxiety) | 1. I've had a lot of headaches, stomach-aches or sickness |
|  |  |  | 2. I've worried a lot |
|  |  |  | 3. I have often been unhappy, down-hearted or tearful |
|  |  |  | 4. I've been nervous in new situations. I've easily lost confidence |
|  |  |  | 5. I've had many fears, I've been easily scared |
|  |  | Peer problems | 1. I have usually been on my own. I have generally kept to myself |
|  |  |  | 2. I have had one good friend or more |
|  |  |  | 3. Other people my age have generally liked me |
|  |  |  | 4. Other people have picked on me or bullied me |
|  |  |  | 5. I have got on better with older adults than with people my own age |
|  |  | Hyperactivity | 1. I have been restless, I've not been able to stay still for long |
|  |  |  | 2. I have constantly been fidgeting or squirming |
|  |  |  | 3. I've been easily distracted, I've found it difficult to concentrate |
|  |  |  | 4. I've thought before I've done things |
|  |  |  | 5. I've finished the work I have been doing. My attention has been good |
|  |  | Conduct problems | 1. I have been very angry and often lost my temper |
|  |  |  | 2. I have usually done as I am told |
|  |  |  | 3. I have fought a lot. I could make other people do what I wanted |
|  |  |  | 4. I have often been accused of lying or cheating |
|  |  |  | 5. I have taken things that are not mine |
|  | Short Mood and Feeling Questionnaire ^8^ | | Please rate how true the following statements have been about you in the past two weeks. Response options: Not true (0), Quite true (1), Very true (2) |
|  |  |  | 1. I felt miserable or unhappy |
|  |  |  | 2. I felt so tired I just sat around and did nothing |
|  |  |  | 3. I was very restless |
|  |  |  | 4. I cried a lot |
|  |  |  | 5. I found it hard to think properly or concentrate |
|  |  |  | 6. I hated myself |
|  |  |  | 7. I felt lonely |
|  |  |  | 8. I thought I could never be as good as other people |
|  | Brief Aggression Questionnaire ^9^ | |  |
|  |  | Physical aggression | 1. Given enough provocation, I may hit another person |
|  |  |  | 2. If I have to resort to violence to protect my rights, I will |
|  |  |  | 3. There are people who pushed me so far that we came to blows |
|  |  | Verbal aggression | 1. I tell my friends openly when I disagree with them |
|  |  |  | 2. When people annoy me, I may tell them what I think of them |
|  |  |  | 3. My friends say that I'm somewhat argumentative |
|  |  | Anger | 1. Sometimes I fly off the handle for no good reason |
|  |  |  | 2. I have trouble controlling my temper |
|  | Antisocial Behavior: Edinburgh Study of Youth Transitions and Crime ^10^ | | During the last year... Response options: Not in the last year or never (0), Once (1), 2 or more times (2) |
|  |  |  | How often have you been rowdy or rude in a public place, so that people complained or you got into trouble? |
|  |  |  | How often have you stolen something from a shop? |
|  |  |  | How often have you bought smt you knew or suspected was stolen? |
|  |  |  | How often have you taken and/or driven a vehicle without the owner's permission? |
|  |  |  | How often have you hit, kicked or punched someone else on purpose, with the intention of really hurting them? |
|  |  |  | How often have you deliberately damaged or destroyed property that did not belong to you? |
|  |  |  | How often have you hurt or injured animals or birds on purpose? |
|  |  |  | How often have you carried a knife or other weapon with you for protection, or in case it was needed in a fight? |
|  |  |  | How often have you used a cheque book, credit card or cash point card which you knew or suspected to be stolen, to get money out of a bank account or to purchase something? |
|  |  |  | How many times did you sell an illegal drug to someone? |
|  |  |  | How many times did you set fire or try to set fire to something on purpose (e.g., a school, bus shelter, house, etc)? |
|  | Conners Hyperactivity Scale ^11^ | | To what extent do the following statements accurately describe you? Not at all true (0), Somewhat true (1), Mainly true (2), Definitely true (3) |
|  |  | Inattention | 1. It is hard for me to pay attention to details |
|  |  |  | 2. I make mistakes by accident |
|  |  |  | 3. I have trouble keeping my mind on what I am doing |
|  |  |  | 4. I have trouble keeping my mind on what other people are saying to me |
|  |  |  | 5. I have trouble following instructions |
|  |  |  | 6. I have trouble finishing things |
|  |  |  | 7. I have trouble keeping myself organised |
|  |  |  | 8. I do not like doing things that make me think hard |
|  |  |  | 9. I lose stuff that I need |
|  |  |  | 10. I get distracted by things that are going on around me |
|  |  |  | 11. I forget stuff |
|  |  | Hyperactivity | 1. It is hard for me to sit still |
|  |  |  | 2. I get out of my seat when I am not supposed to |
|  |  |  | 3. I am restless |
|  |  |  | 4. I have trouble doing things quietly |
|  |  |  | 5. I like to be on the go rather than being in one place |
|  |  |  | 6. I talk too much |
|  |  |  | 7. I blurt out the answer before a question is finished |
|  |  |  | 8. I have trouble waiting for my turn |
|  |  |  | 9. I interrupt other people |
|  | Specific Psychotic Experiences Questionnaire ^12^ | | Please indicate how often you have had the thought or feeling described. Response options: Not at all (0), Rarely (1), Once a month (2), Once a week (3), Several times a week (4), Daily (5) |
|  |  | Paranoia | 1. Someone has bad intentions towards me |
|  |  |  | 2. Bad things are being said about me behind my back |
|  |  |  | 3. People are being hostile towards me |
|  |  |  | 4. People are trying to upset me |
|  |  |  | 5. Someone has it in for me |
|  |  |  | 6. People are looking at me in an unfriendly way |
|  |  |  | 7. There might be negative comments being spread about me |
|  |  |  | 8. People might be conspiring against me |
|  |  |  | 9. I am under threat from others |
|  |  |  | 10. People are laughing at me |
|  |  |  | 11. People would harm me if given an opportunity |
|  |  |  | 13. I need to be on my guard against others |
|  |  |  | 14. I might be being observed or followed |
|  |  |  | 15. I can detect coded messages about me in the press/TV/internet |
|  |  | Hallucinations | 1. I hear sounds or music that people near me don't hear |
|  |  |  | 2. I see things that other people cannot |
|  |  |  | 3. I feel that someone is touching me, but when I look nobody is there |
|  |  |  | 4. I hear noises or sounds when there is nothing about to explain them |
|  |  |  | 5. I detect smells which don't seem to come from my surroundings |
|  |  |  | 6. I see shapes, lights, or colours even though there is nothing really there |
|  |  |  | 7. I notice smells or odours that people next to me seem unaware of |
|  |  |  | 8. I experience unusual burning sensations or other strange feelings in or on my body that can't be explained |
|  |  |  | 9. I hear voices commenting on what I'm thinking or doing |
| Subjective well-being | |  |  |
|  | Contentment with Life Assessment Scale ^13^ | | How much do you agree with the following statements? Strongly disagree (1), Disagree (2), Neither agree nor disagree (3), Agree (4), Strongly agree (5) |
|  |  | Love and Relationships | 1. I am happy with my love life |
|  |  |  | 2. I have the level of intimacy in my life that I want |
|  |  |  | 3. I feel loved |
|  |  | Community | 1. I enjoy living in my neighbourhood |
|  |  |  | 2. I would prefer to move to a different area if I could |
|  |  |  | 3. I feel a sense of belonging in my neighbourhood |
|  |  |  | 4. I feel like an outsider in my neighbourhood |
|  |  |  | 5. I feel safe in my neighbourhood |
|  |  | Financial Wellbeing | 1. I am able to save enough money for holidays or other luxuries |
|  |  |  | 2. I feel I am doing reasonably well financially |
|  |  |  | 3. I rarely need to ask for outside help in meeting my expenses |
|  |  |  | 4. I can afford to treat myself now and then if I feel like it |
|  |  |  | 5. I sometimes struggle to pay my bills |

*Note*. The measures of self-control (the Brief Self-Control Measure and the Consideration of Future Consequences Scale) were reverse-coded for analyses, such that higher scores reflected more self-control, except when estimating latent factors.

## Supplementary Materials 1. Lived-experience contribution

This study was informed by a parallel co-produced qualitative investigation involving individuals with lived experience of childhood maltreatment and intimate partner violence victimization ^14^. We conducted semi-structured interviews with sixteen adults from the UK to explore the psychological processes they perceived as linking maltreatment to subsequent IPV victimization. Full details of the qualitative methodology and findings are reported in ^14^.

Lived-experience input influenced the current study in three ways. First, it guided our choice of candidate mediators. For example, participants described seeking relationships to fulfil unmet childhood needs, a form of risk-taking that could inadvertently increase vulnerability to unsafe relationships. Second, it guided interpretation. For example, participants described how childhood maltreatment undermined their self-worth – a core aspect of subjective well-being – which in turn disrupted their ability to form safe relationships by leading them to tolerate or remain in harmful relationships. Third, it shaped our discussion of intervention strategies, as participants stressed the importance of approaches that address the psychological pathways connecting maltreatment and IPV, while recognising broader relational and social contexts.

## Supplementary Materials 2. Data preparation

Data preparation was conducted in R ^15^ using the packages dplyr ^16^, psych ^17^, naniar ^18^, mice ^19^, and gee ^20^. First, we inspected the distribution of the composite (mean) scores and polygenic scores (PGS) available in TEDS ([Supplementary Table 2](#_Supplementary_Table_2.)). Skewness and kurtosis values were within the normal range (-3–3 and -10–10, respectively), per structural equation modeling guidelines ^21^ except for antisocial behavior, which was leptokurtic. We addressed this by using robust estimation methods in statistical analyses. The phenotypic composite scores varied in terms of internal consistency and intraclass reliability, but all demonstrated a single factor underlying their items ([Supplementary Table 3](#_Table_4)). The pattern of missingness was not completely random, as indicated by a significant Little’s Missing Completely at Random test ^22^, *χ^2^* (16870) = 28660.59, *p* < .001. Inspecting frequencies revealed that more data points were missing for variables collected in a second wave of the TEDS 21 data collection (i.e., childhood maltreatment, crime, peer victimization, psychotic experiences, and ADHD). We imputed composite scores using the Multiple Imputation by Chained Equations method with 30 iterations ^23^, which converged adequately, as indicated by stable summary statistics (e.g., median discrepancy in mean values between raw and imputed data = 0.04). All measures were z-standardized before statistical analyses.

### Supplementary Table 2 (ST2). Distribution of raw phenotypic composite scores and polygenic scores

|  | n | mean | sd | se | median | trimmed | mad | min | max | range | skew | kurtosis |
| --- | --- | --- | --- | --- | --- | --- | --- | --- | --- | --- | --- | --- |
| Phenotypic composite scores |  |  |  |  |  |  |  |  |  |  |  |  |
| Childhood maltreatment | 8368 | 5.30 | 4.55 | 0.05 | 4.00 | 4.54 | 2.97 | 0.00 | 32.00 | 32.00 | 2.06 | 5.86 |
| Intimate partner violence | 8958 | 1.76 | 0.96 | 0.01 | 1.33 | 1.59 | 0.49 | 1.00 | 5.00 | 4.00 | 1.37 | 1.25 |
| Neuroticism | 8925 | 2.62 | 0.66 | 0.01 | 2.67 | 2.61 | 0.74 | 1.00 | 5.00 | 4.00 | 0.20 | -0.21 |
| Extraversion | 8919 | 3.51 | 0.65 | 0.01 | 3.50 | 3.55 | 0.74 | 1.00 | 5.00 | 4.00 | -0.49 | 0.05 |
| Openness | 8911 | 3.41 | 0.62 | 0.01 | 3.33 | 3.41 | 0.74 | 1.00 | 5.00 | 4.00 | -0.02 | -0.21 |
| Agreeableness | 8904 | 3.71 | 0.57 | 0.01 | 3.83 | 3.74 | 0.49 | 1.00 | 5.00 | 4.00 | -0.49 | 0.48 |
| Conscientiousness | 8899 | 3.88 | 0.62 | 0.01 | 4.00 | 3.92 | 0.49 | 1.00 | 5.00 | 4.00 | -0.72 | 0.77 |
| Self-control | 8893 | -9.56 | 4.19 | 0.04 | -9.00 | -9.39 | 4.45 | -24.00 | 0.00 | 24.00 | -0.38 | -0.18 |
| Consideration of future consequences | 8890 | 2.46 | 0.93 | 0.01 | 2.25 | 2.41 | 1.11 | 1.00 | 5.00 | 4.00 | 0.42 | -0.59 |
| Risk-taking | 8889 | 6.50 | 3.58 | 0.04 | 6.00 | 6.29 | 2.97 | 0.00 | 23.00 | 23.00 | 0.57 | 0.16 |
| Anxiety | 9141 | 3.60 | 2.69 | 0.03 | 3.00 | 3.41 | 2.97 | 0.00 | 10.00 | 10.00 | 0.50 | -0.70 |
| Peer problems | 9141 | 2.17 | 1.80 | 0.02 | 2.00 | 1.98 | 1.48 | 0.00 | 10.00 | 10.00 | 0.90 | 0.73 |
| Hyperactivity | 9140 | 3.30 | 2.19 | 0.02 | 3.00 | 3.18 | 2.97 | 0.00 | 10.00 | 10.00 | 0.50 | -0.26 |
| Conduct problems | 9142 | 1.58 | 1.32 | 0.01 | 1.00 | 1.42 | 1.48 | 0.00 | 9.00 | 9.00 | 1.56 | 3.66 |
| Depressive symptoms | 9138 | 4.45 | 4.11 | 0.04 | 3.00 | 3.90 | 2.97 | 0.00 | 16.00 | 16.00 | 0.98 | 0.15 |
| Aggression | 8886 | 2.52 | 0.77 | 0.01 | 2.50 | 2.49 | 0.74 | 1.00 | 5.00 | 4.00 | 0.39 | -0.26 |
| Antisocial behavior | 8111 | 0.22 | 0.64 | 0.01 | 0.00 | 0.07 | 0.00 | 0.00 | 9.00 | 9.00 | 4.36 | 27.74 |
| Attention-deficit hyperactivity disorder | 8323 | 13.63 | 8.89 | 0.10 | 12.00 | 12.66 | 7.41 | 0.00 | 60.00 | 60.00 | 1.15 | 1.76 |
| Psychotic experiences | 8318 | 5.90 | 6.86 | 0.08 | 3.50 | 4.61 | 4.45 | 0.00 | 55.50 | 55.50 | 2.18 | 6.44 |
| Subjective well-being | 8907 | 3.61 | 0.61 | 0.01 | 3.67 | 3.64 | 0.63 | 1.24 | 5.00 | 3.76 | -0.36 | -0.07 |
| Polygenic scores |  |  |  |  |  |  |  |  |  |  |  |  |
| Childhood maltreatment | 8406 | -0.04 | 0.13 | 0.00 | -0.03 | -0.04 | 0.13 | -0.49 | 0.41 | 0.90 | 0.02 | -0.10 |
| Conscientiousness | 8406 | 0.61 | 0.04 | 0.00 | 0.61 | 0.61 | 0.04 | 0.45 | 0.78 | 0.33 | 0.01 | 0.07 |
| Openness | 8406 | -0.39 | 0.04 | 0.00 | -0.39 | -0.39 | 0.04 | -0.58 | -0.24 | 0.34 | 0.01 | 0.07 |
| Irritability | 8406 | 0.41 | 0.14 | 0.00 | 0.41 | 0.41 | 0.14 | -0.12 | 0.87 | 0.99 | 0.02 | 0.11 |
| Neuroticism | 8406 | -0.07 | 0.21 | 0.00 | -0.07 | -0.07 | 0.21 | -0.94 | 0.67 | 1.61 | 0.00 | 0.07 |
| Risk-taking (sexual partners) | 8406 | 0.44 | 0.19 | 0.00 | 0.44 | 0.44 | 0.19 | -0.43 | 1.20 | 1.63 | 0.00 | -0.01 |
| Risk-taking (first PC) | 8406 | 1.04 | 0.21 | 0.00 | 1.04 | 1.04 | 0.20 | 0.19 | 1.84 | 1.65 | -0.09 | 0.07 |
| Risk tolerance | 8406 | 0.42 | 0.12 | 0.00 | 0.42 | 0.42 | 0.12 | -0.08 | 0.88 | 0.96 | -0.01 | -0.02 |
| Externalizing | 8406 | 0.17 | 0.17 | 0.00 | 0.16 | 0.17 | 0.17 | -0.44 | 0.79 | 1.23 | 0.00 | -0.04 |
| Aggression | 8406 | 0.05 | 0.05 | 0.00 | 0.05 | 0.05 | 0.05 | -0.14 | 0.24 | 0.38 | 0.01 | 0.11 |
| Depressive symptoms | 8406 | 1.34 | 0.37 | 0.00 | 1.34 | 1.34 | 0.36 | -0.03 | 2.81 | 2.83 | -0.01 | 0.02 |
| Anxiety | 8406 | 1.38 | 0.38 | 0.00 | 1.37 | 1.38 | 0.39 | -0.02 | 2.92 | 2.94 | 0.03 | -0.05 |
| Schizophrenia | 8406 | 7.12 | 0.97 | 0.01 | 7.13 | 7.12 | 0.96 | 3.65 | 10.48 | 6.83 | -0.03 | -0.03 |
| Attention-deficit hyperactivity disorder | 8406 | 1.05 | 0.56 | 0.01 | 1.05 | 1.05 | 0.56 | -0.97 | 2.91 | 3.89 | 0.00 | 0.03 |
| Subjective well-being | 8406 | 0.19 | 0.11 | 0.00 | 0.19 | 0.19 | 0.11 | -0.20 | 0.57 | 0.77 | 0.01 | -0.02 |

*Note*: Response options and corresponding values are reported in Supplementary Table 1. The composite scores of childhood maltreatment, depressive symptoms, attention-deficit hyperactivity disorder, and psychotic experiences were computed as the mean multiplied by the number of component items.

### Supplementary Table 3 (ST3). Psychometric properties of raw composite scores

|  | 𝛼 | ɷ_t_ | ICC | χ^2^ | df | *p* |
| --- | --- | --- | --- | --- | --- | --- |
| Childhood maltreatment | 0.90 | 0.91 | 0.75 | 4575.66 | 20.00 | < 0.001 |
| Intimate partner violence | 0.91 | 0.93 | 0.89 | 3740.49 | 9.00 | < 0.001 |
| Neuroticism | 0.67 | 0.74 | 0.62 | 782.21 | 9.00 | < 0.001 |
| Extraversion | 0.69 | 0.75 | 0.66 | 580.27 | 9.00 | < 0.001 |
| Openness | 0.61 | 0.68 | 0.46 | 539.44 | 9.00 | < 0.001 |
| Agreeableness | 0.63 | 0.69 | 0.55 | 382.63 | 9.00 | < 0.001 |
| Conscientiousness | 0.74 | 0.79 | 0.73 | 535.91 | 9.00 | < 0.001 |
| Self-control | 0.69 | 0.74 | 0.65 | 205.61 | 9.00 | < 0.001 |
| Consideration of future consequences | 0.84 | 0.88 | 0.84 | 89.16 | 2.00 | < 0.001 |
| Risk-taking | 0.61 | 0.68 | 0.51 | 521.71 | 9.00 | < 0.001 |
| Anxiety | 0.79 | 0.83 | 0.74 | 274.98 | 5.00 | < 0.001 |
| Peer problems | 0.57 | 0.66 | 0.47 | 145.45 | 5.00 | < 0.001 |
| Hyperactivity | 0.66 | 0.74 | 0.65 | 1262.88 | 5.00 | < 0.001 |
| Conduct problems | 0.48 | 0.59 | 0.12 | 108.61 | 5.00 | < 0.001 |
| Depressive symptoms | 0.87 | 0.89 | 0.86 | 1536.99 | 20.00 | < 0.001 |
| Aggression | 0.80 | 0.82 | 0.73 | 8388.02 | 20.00 | < 0.001 |
| Antisocial behavior | 0.56 | 0.60 | 0.53 | 650.90 | 44.00 | < 0.001 |
| Psychotic symptoms | 0.58 | 0.79 | 0.04 | 1532.73 | 1.00 | < 0.001 |
| Attention-deficit hyperactivity disorder | 0.65 | 0.83 | 0.56 | 2204.11 | 1.00 | < 0.001 |
| Subjective well-being | 0.34 | 0.56 | 0.31 | 579.00 | 3.00 | < 0.001 |

*Note*: 𝛼 = Cronbach's alpha; ɷ_t_ = omega total, computed as the amount of reliable variance in the variables accounted for by a single factor ^24^; ICC = intraclass correlation coefficient, computed as per the two-way random effects model with single measures approach ^25^; *χ*^2^ = chi-square test statistic for the Confirmatory Factor Analysis (CFA) models extracting a single factor from the selected items, estimated using default parameters in lavaan ^26^; df = degrees of freedom and *p* = *p*-value associated with the *χ*^2^ test comparing each CFA model with their baseline models representing the null hypothesis that items are uncorrelated. Despite low internal consistency (α = 0.34), which would typically attenuate observed associations, subjective well-being showed particularly strong across subsequent statistical analyses, suggesting a robust association with maltreatment and IPV victimization.

## Supplementary Materials 3. Generalized Estimating Equations

### Methods

We fitted a series of univariate generalized estimating equations (GEE ^20^) with the R package geepack ^27^ to examine phenotypic associations between victimization experiences (maltreatment and IPV victimization) and between each of these experiences and the range of personality and mental health phenotypes we selected as candidate mediators based on lived-experience input and prior research. In the first set of univariate models, we specified maltreatment as the predictor, and IPV victimization and the personality and mental health phenotypes as outcomes. Next, we specified maltreatment and the personality and mental health phenotypes as predictors and IPV as the outcome. We accounted for the non-independence of observations (i.e., twins within families) by specifying an exchangeable correlation structure using a family identifier as the clustering variable. We applied Bonferroni correction ^28^ to the estimated *p*-values to adjust for multiple comparisons.

We then examined the associations between the PGS for our candidate mediators and the respective experiences and phenotypes in TEDS, using GEE. For this, we used the PGSs for childhood maltreatment ^29^, neuroticism ^30^, openness and conscientiousness ^31^, risk tolerance and risk taking ^32^, anxiety ^33^, depression ^34^, aggression ^35^, externalizing symptoms ^36,37^, schizophrenia ^38^, ADHD ^39^, and subjective well-being ^40^ available in TEDS. We included the first 10 principal components, chip plates used, batches, sex, and age as covariates, we z-standardized PGSs, and applied Bonferroni correction.

### Results

Results of GEE analyses with phenotypes are reported in [Supplementary Tables 4 and 5](#_Supplementary_Table_4.). We estimated a moderate significant association between maltreatment and IPV victimization. Both experiences were significantly associated with small-to-moderate effect sizes with each of the personality and mental health phenotypes, except for openness, which was therefore not examined in mediation analyses. Maltreatment showed the strongest positive associations with psychotic experiences, ADHD, and depressive symptoms, and the strongest negative associations with self-control, subjective well-being, and the personality traits of conscientiousness and agreeableness. IPV victimization showed the strongest positive associations with psychotic experiences, depressive symptoms, anxiety, peer problems and conduct problems, and the strongest negative associations with subjective well-being, self-control, and conscientiousness.

Results of GEE analyses with PGSs are reported in [Supplementary Table 6](#_Table_5). We found that all PGSs except for the PGS for conscientiousness and schizophrenia were significantly associated with the respective phenotypes in TEDS; these two PGSs were thus excluded from PGS regression analyses. For risk-taking – the phenotype with multiple corresponding PGSs in TEDS, we retained the most strongly associated PGS, namely the PGS for risk tolerance.

#### Supplementary Table 4 (ST4). Generalized Estimating Equations results, phenotypic associations with maltreatment

|  | Predictor | | | | |
| --- | --- | --- | --- | --- | --- |
|  | Childhood maltreatment | | | | |
| Outcome | Estimate | lCI | uCI | *p* | *p__adj_* |
| Intimate partner violence victimization | 0.23 | 0.21 | 0.25 | < 0.001 | < 0.001 |
| Neuroticism | 0.18 | 0.16 | 0.20 | < 0.001 | < 0.001 |
| Extraversion | -0.11 | -0.13 | -0.09 | < 0.001 | < 0.001 |
| Openness | 0.02 | 0.00 | 0.04 | 0.077 | 1.000 |
| Agreeableness | -0.13 | -0.15 | -0.10 | < 0.001 | < 0.001 |
| Conscientiousness | -0.13 | -0.15 | -0.11 | < 0.001 | < 0.001 |
| Self-control | -0.19 | -0.21 | -0.17 | < 0.001 | < 0.001 |
| Consideration of future consequences | -0.07 | -0.10 | -0.05 | < 0.001 | < 0.001 |
| Risk-taking | 0.15 | 0.13 | 0.17 | < 0.001 | < 0.001 |
| Anxiety | 0.19 | 0.17 | 0.21 | < 0.001 | < 0.001 |
| Peer problems | 0.18 | 0.16 | 0.21 | < 0.001 | < 0.001 |
| Hyperactivity | 0.20 | 0.17 | 0.22 | < 0.001 | < 0.001 |
| Conduct problems | 0.17 | 0.15 | 0.20 | < 0.001 | < 0.001 |
| Depressive symptoms | 0.24 | 0.22 | 0.26 | < 0.001 | < 0.001 |
| Aggression | 0.19 | 0.17 | 0.21 | < 0.001 | < 0.001 |
| Antisocial behavior | 0.20 | 0.17 | 0.22 | < 0.001 | < 0.001 |
| ADHD | 0.25 | 0.23 | 0.28 | < 0.001 | < 0.001 |
| Psychotic experiences | 0.40 | 0.37 | 0.43 | < 0.001 | < 0.001 |
| Subjective well-being | -0.18 | -0.20 | -0.16 | < 0.001 | < 0.001 |

*Note*: Standardized estimates. lCI = 95% confidence intervals, lower bound; uCI = 95% confidence intervals, upper bound; *p__adj_* = Bonferroni-adjusted *p* value.

#### Supplementary Table 5 (ST5). Generalized Estimating Equations results, phenotypic associations with intimate partner violence victimization

|  | Outcome | | | | |
| --- | --- | --- | --- | --- | --- |
|  | Intimate partner violence victimization | | | | |
| Predictor | Estimate | lCI | uCI | *p* | *p_adj* |
| Neuroticism | 0.19 | 0.17 | 0.21 | < 0.001 | < 0.001 |
| Extraversion | -0.08 | -0.10 | -0.06 | < 0.001 | < 0.001 |
| Openness | 0.02 | 0.00 | 0.05 | 0.017 | 0.305 |
| Agreeableness | -0.07 | -0.09 | -0.05 | < 0.001 | < 0.001 |
| Conscientiousness | -0.12 | -0.14 | -0.10 | < 0.001 | < 0.001 |
| Self-control | -0.17 | -0.19 | -0.15 | < 0.001 | < 0.001 |
| Consideration of future consequences | -0.09 | -0.11 | -0.07 | < 0.001 | < 0.001 |
| Risk-taking | 0.13 | 0.11 | 0.15 | < 0.001 | < 0.001 |
| Anxiety | 0.23 | 0.21 | 0.25 | < 0.001 | < 0.001 |
| Peer problems | 0.23 | 0.21 | 0.25 | < 0.001 | < 0.001 |
| Hyperactivity | 0.21 | 0.19 | 0.23 | < 0.001 | < 0.001 |
| Conduct problems | 0.23 | 0.21 | 0.26 | < 0.001 | < 0.001 |
| Depressive symptoms | 0.26 | 0.24 | 0.28 | < 0.001 | < 0.001 |
| Aggression | 0.18 | 0.16 | 0.20 | < 0.001 | < 0.001 |
| Antisocial behavior | 0.14 | 0.12 | 0.16 | < 0.001 | < 0.001 |
| ADHD | 0.20 | 0.18 | 0.22 | < 0.001 | < 0.001 |
| Psychotic experiences | 0.26 | 0.24 | 0.28 | < 0.001 | < 0.001 |
| Subjective well-being | -0.21 | -0.23 | -0.19 | < 0.001 | < 0.001 |

*Note*: Standardized estimates. lCI = 95% confidence intervals, lower bound; uCI = 95% confidence intervals, upper bound; *p* = *p* value; *p__adj_* = Bonferroni-adjusted *p* value.

#### Supplementary Table 6 (ST6). Polygenic score instrument diagnostics

| Polygenic score | Phenotype in TEDS | Discovery GWAS | Discovery N | *h^2^* | *h^2^*SE | Z-score | SNPs in PGS | Δ*R^2^* | F-statistic | *β* | lCI | uCI | *p* | *p__adj_* |
| --- | --- | --- | --- | --- | --- | --- | --- | --- | --- | --- | --- | --- | --- | --- |
| Childhood maltreatment | Childhood maltreatment | Warrier et al., 2021 | 185,414 | 0.079 | 0.0042 | 18.8 | 1,162,498 | 0.01 | 65.44 | 0.10 | 0.07 | 0.13 | < 0.001 | < 0.001 |
| Neuroticism | Neuroticism | Nagel et al., 2018 | 390,278 | 0.01 | 0.003 | 33.3 | 1,090,633 | 0.01 | 65.21 | 0.11 | 0.08 | 0.14 | < 0.001 | < 0.001 |
| Openness | Openness | De Moor et al., 2012 | 17,375 | 0.106 | 0.0267 | 4 | 436,342 | 0.00 | 19.47 | 0.06 | 0.03 | 0.09 | < 0.001 | < 0.001 |
| Conscientiousness | Conscientiousness | De Moor et al., 2012 | 17,375 | 0.0728 | 0.0297 | 2.5 | 497,525 | 0.00 | 2.30 | 0.02 | -0.01 | 0.05 | 0.139 | 1.000 |
| Risk-taking (first PC) | Risk-taking | Karlsson Linnér et al., 2019 | 315,894 | 0.156 | 0.004 | 39 | 500,760 | 0.02 | 100.30 | 0.13 | 0.10 | 0.16 | < 0.001 | < 0.001 |
| Risk tolerance | Risk-taking | Karlsson Linnér et al., 2019 | 466,571 | 0.045 | 0.001 | 45 | 500,760 | 0.02 | 113.54 | 0.15 | 0.12 | 0.17 | < 0.001 | < 0.001 |
| Risk-taking (sexual partners) | Risk-taking | Karlsson Linnér et al., 2019 | 370,711 | 0.128 | 0.003 | 42.7 | 500,760 | 0.01 | 63.68 | 0.11 | 0.08 | 0.13 | < 0.001 | < 0.001 |
| Anxiety | Anxiety | Purves et al., 2020 | 84,217 | 0.034 | 0.031 | 10.5 | 867,317 | 0.01 | 37.47 | 0.08 | 0.05 | 0.11 | < 0.001 | < 0.001 |
| Depression | Depressive symptoms | McIntosh et al., 2024 | 2,000,702 | 0.084 | 0.0007 | 120 | 1,125,232 | 0.02 | 100.31 | 0.13 | 0.10 | 0.16 | < 0.001 | < 0.001 |
| Aggression | Aggression | Ip et al., 2021 | 80,931 | 0.0331 | 0.0038 | 8.7 | 906,378 | 0.01 | 34.62 | 0.08 | 0.05 | 0.11 | < 0.001 | < 0.001 |
| Externalizing | Conduct problems | Karlsson Linnér et al., 2021 | 1,045,957 | varies | varies | varies | 973,312 | 0.01 | 89.61 | 0.13 | 0.10 | 0.15 | < 0.001 | < 0.001 |
| Externalizing symptoms | Antisocial behavior | Karlsson Linnér et al., 2021 | 1,045,957 | varies | varies | varies | 973,312 | 0.01 | 75.03 | 0.11 | 0.08 | 0.14 | < 0.001 | < 0.001 |
| Attention-deficit hyperactivity disorder | Hyperactivity | Demontis et al., 2023 | 47,382 | 0.2398 | 0.0151 | 15.9 | 1,069,013 | 0.01 | 50.48 | 0.09 | 0.06 | 0.12 | < 0.001 | < 0.001 |
| Attention-deficit hyperactivity disorder | Attention-deficit hyperactivity disorder | Demontis et al., 2023 | 47,382 | 0.2398 | 0.0151 | 15.9 | 1,069,013 | 0.01 | 48.51 | 0.09 | 0.06 | 0.12 | < 0.001 | < 0.001 |
| Schizophrenia | Psychotic experiences | Trubetskoy et al., 2022 | 130644 | 0.24 | 0.0068 | 35.3 | 1,100,223 | 0.00 | 6.28 | 0.03 | 0.00 | 0.05 | 0.038 | 1.000 |
| Subjective well-being | Subjective well-being | Okbay et al., 2016 | 298,420 |  |  |  | 427,033 | 0.00 | 17.58 | 0.05 | 0.03 | 0.08 | < 0.001 | 0.004 |

*Note*. GWAS = genome-wide association study; TEDS = Twins Early Development Study; SNPs = single nucleotide polymorphisms; Δ*R^2^* = proportion of variance explained by the polygenic score in TEDS beyond the covariates (first 10 principal components, chip plates used, batches, sex, and age). Δ*R^2^* and F-statistics were obtained from ordinary least squares (OLS) regressions regressing the phenotype measured in TEDS on the corresponding PGS in a subset of the sample consisting of one randomly selected twin per family (*n* = 6521). All F-statistics substantially exceed the conventional threshold of 10 for strong instruments^41^). *β* = Standardized beta coefficient; lCI = 95% confidence intervals, lower bound; uCI = 95% confidence intervals, upper bound; *p* = *p* value; *p__adj_* = Bonferroni-adjusted *p* value. Beta coefficients and corresponding confidence intervals and p values are from Generalized Estimating Equations (GEE) in the full sample, accounting for non-independence of observations.

## Supplementary Materials 4. Factor analysis

### Methods

We conducted factor analysis ^21^ using the R package lavaan ^26^ to extract latent factors for subsequent mediation analyses. First, we reverse-coded variables negatively associated with psychopathology, including conscientiousness, agreeableness, and subjective well-being, ensuring that higher values reflected lower levels of these traits. Openness was included in the factor analysis without reverse-coding, as prior research suggests its positive associations with psychopathology (mania and thought disorder ^42,43^). We then assessed factorability using the Kaiser-Meyer-Olkin (KMO) test and determined the optimal number of factors through parallel analysis. We performed Exploratory Factor Analysis (EFA) with oblimin rotation, extracting a decreasing number of factors, with items assigned to their strongest-loading factor. We then used Confirmatory Factor Analysis (CFA) to compare the factor structures emerged from EFA. We conducted both EFA and CFA in the full sample rather than splitting the data, as split-sample approaches reduce statistical power and are not universally regarded as best practice to prevent overfitting, particularly relative to comparing alternative factor structures ^21,44,45^. We used a family identifier as the clustering variable to account for the non-independence of observations.

### Results

The KMO test indicated that the data were highly suitable for factor analysis (overall Measure of Sampling Adequacy [MSA] = 0.87) and parallel analysis suggested six factors. CFA indicated that the five-factor model provided the best fit; however, one factor comprised only two items, which is not recommended per SEM guidelines ^21^. Therefore, we retained the two-factor model, which had the next best theoretically meaningful structure with acceptable absolute fit indices (CLI and TLI) and provided a parsimonious and theoretically meaningful structure (factor loadings in [Supplementary Table 7](#_Supplementary_Table_6._1), model fit indices in [Supplementary Table 8](#_Supplementary_Table_6._1)). The two factors broadly corresponded to negative/disordered affect (Factor 1) and externalizing tendencies (Factor 2).

#### Supplementary Table 7 (ST7). Factor loadings, phenotypic and genetically informed latent factor models

|  | CFA | | | SEM | | | MR-DoC | | |
| --- | --- | --- | --- | --- | --- | --- | --- | --- | --- |
|  | Estimate | lCI | uCI | Estimate | lCI | uCI | Estimate | lCI | uCI |
| Negative/disordered affect | | | | | | | | | |
| Neuroticism | 0.72 | 0.69 | 0.75 | 0.71 | 0.69 | 0.72 | 0.74 | 0.73 | 0.75 |
| Extraversion | 0.59 | 0.56 | 0.62 | 0.45 | 0.43 | 0.47 | 0.47 | 0.46 | 0.48 |
| Anxiety | 0.84 | 0.82 | 0.86 | 0.79 | 0.79 | 0.80 | 0.82 | 0.82 | 0.83 |
| Peer problems | 0.59 | 0.56 | 0.62 | 0.53 | 0.52 | 0.54 | 0.56 | 0.54 | 0.57 |
| Depressive symptoms | 0.75 | 0.73 | 0.77 | 0.79 | 0.78 | 0.80 | 0.84 | 0.84 | 0.85 |
| Psychotic experiences | 0.39 | 0.35 | 0.43 | 0.50 | 0.48 | 0.51 | 0.53 | 0.51 | 0.54 |
| Subjective well-being | 0.40 | 0.37 | 0.43 | 0.45 | 0.42 | 0.47 | 0.49 | 0.48 | 0.50 |
| Externalizing tendencies | | | | | | | | | |
| Openness | 0.14 | 0.11 | 0.17 | 0.12 | 0.10 | 0.14 | 0.13 | 0.12 | 0.14 |
| Agreeableness | 0.42 | 0.39 | 0.45 | 0.33 | 0.31 | 0.35 | 0.34 | 0.32 | 0.35 |
| Conscientiousness | 0.53 | 0.50 | 0.56 | 0.60 | 0.57 | 0.60 | 0.61 | 0.60 | 0.61 |
| Self-control | 0.66 | 0.64 | 0.68 | 0.74 | 0.72 | 0.74 | 0.79 | 0.78 | 0.79 |
| Consideration of future consequences | 0.47 | 0.44 | 0.50 | 0.47 | 0.45 | 0.48 | 0.49 | 0.48 | 0.50 |
| Risk-taking | 0.67 | 0.64 | 0.70 | 0.42 | 0.41 | 0.44 | 0.42 | 0.41 | 0.43 |
| Hyperactivity | 0.51 | 0.48 | 0.54 | 0.67 | 0.66 | 0.68 | 0.73 | 0.72 | 0.74 |
| Conduct problems | 0.54 | 0.50 | 0.58 | 0.51 | 0.49 | 0.53 | 0.52 | 0.51 | 0.53 |
| Aggression | 0.52 | 0.49 | 0.55 | 0.44 | 0.43 | 0.45 | 0.46 | 0.45 | 0.47 |
| Antisocial behavior | 0.39 | 0.35 | 0.43 | 0.30 | 0.29 | 0.33 | 0.31 | 0.29 | 0.32 |
| ADHD | 0.56 | 0.53 | 0.59 | 0.65 | 0.64 | 0.65 | 0.70 | 0.69 | 0.71 |

*Note*: Standardized estimates. ADHD = Attention-deficit Hyperactivity Disorder; lCI = 95% Confidence Intervals, lower bound, uCI = 95% Confidence Intervals, upper bound; CFA = Confirmatory Factor Analysis (phenotypic); SEM = Structual Equation Modeling (phenotypic); MR-DoC = Mendelian Randomization Direction of Causation Model (genetically informed). The composite scores for extraversion, conscientiousness, agreeableness, and subjective well-being were reverse-coded before factor analysis, such that higher scores reflected higher psychiatric risk.

#### Supplementary Table 8 (ST8). Model fit indices, confirmatory factor analyses

| Factors | AIC | BIC | CFI | TLI | RMSEA | SRMR |
| --- | --- | --- | --- | --- | --- | --- |
| 6 | 523302.20 | 523808.40 | 0.786 | 0.727 | 0.106 | 0.079 |
| 5 | 521326.30 | 521795.80 | 0.813 | 0.772 | 0.097 | 0.070 |
| 4 | 523767.90 | 524200.70 | 0.779 | 0.740 | 0.103 | 0.078 |
| 3 | 523760.30 | 524178.40 | 0.779 | 0.744 | 0.103 | 0.075 |
| 2 | 524948.90 | 525352.40 | 0.763 | 0.729 | 0.106 | 0.078 |
| 1 | 535003.00 | 535399.20 | 0.622 | 0.572 | 0.133 | 0.096 |

*Note*: AIC = Akaike Information Criterion, BIC = Bayesian Information Criterion, CFI = Comparative Fit Index, TLI = Tucker–Lewis Index, RMSEA = Root Mean Square Error of Approximation, SRMR = Standardised Root Mean Square Residual.

## Supplementary Materials 5. Twin modeling

### Methods

We conducted univariate twin analyses ^46^ using the R package TwinAnalysis ^47^ to estimate the latent additive genetic (denoted as ‘A’), shared environmental (‘C’), and nonshared environmental (‘E’) variance components (the latter also capturing measurement error) of the victimization experiences (maltreatment and IPV) and of the personality and mental health phenotypes that were significantly associated with them in GEE. We then used multivariate twin models to estimate the bivariate correlations between the A, C, and E influences (i.e., *r_g_*, *r_c_* and *r_e_*, respectively) on maltreatment, IPV victimization, and associated intermediary phenotypes.

### Results

Univariate twin models indicated small-to-moderate additive genetic influences across victimization experiences, personality, and mental health phenotypes (estimates in [Supplementary Table 9](#_Supplementary_Table_7._2), model fit indices in [Supplementary Table 10](#_Supplementary_Table_8.)). All variables except maltreatment, IPV victimization, anxiety, peer problems, and Factor 1 showed non-significant shared environmental estimates. Maltreatment was the only variable with a substantial proportion of variance attributable to shared environmental factors.

Multivariate twin models (estimates in [Supplementary Table 11](#_Supplementary_Table_10.), model fit indices in [Supplementary Table 12](#_Supplementary_Table_11.)) indicated a substantial genetic correlation between childhood maltreatment and IPV victimization. Both maltreatment and IPV victimization showed the strongest positive genetic correlations with psychotic experiences and externalizing problems (aggression and ASB, respectively) and the strongest negative genetic correlations with subjective well-being and conscientiousness. Psychotic experiences and depressive symptoms showed positive non-shared environmental correlations with both maltreatment and IPV victimization, whereas subjective well-being, agreeableness and conscientiousness showed negative non-shared environmental correlations.

#### Supplementary Table 9 (ST9). Estimates, univariate twin models

|  | A% | lCI | uCI | C% | lCI | uCI | E% | lCI | uCI |
| --- | --- | --- | --- | --- | --- | --- | --- | --- | --- |
| Childhood maltreatment | 0.31 | 0.26 | 0.36 | 0.30 | 0.26 | 0.34 | 0.39 | 0.37 | 0.41 |
| Intimate partner violence victimization | 0.17 | 0.10 | 0.24 | 0.07 | 0.02 | 0.12 | 0.76 | 0.73 | 0.79 |
| Peer victimization | 0.28 | 0.23 | 0.30 | 0.00 | NA | 0.03 | 0.72 | 0.70 | 0.74 |
| Neuroticism | 0.32 | 0.25 | 0.38 | 0.03 | 0.00 | 0.08 | 0.65 | 0.62 | 0.67 |
| Extraversion | 0.39 | 0.37 | 0.42 | 0.00 | NA | 0.01 | 0.61 | 0.58 | 0.63 |
| Openness | 0.36 | 0.33 | 0.38 | 0.00 | NA | 0.02 | 0.64 | 0.62 | 0.67 |
| Agreeableness | 0.24 | 0.21 | 0.26 | 0.00 | NA | 0.01 | 0.76 | 0.74 | 0.79 |
| Conscientiousness | 0.30 | 0.28 | 0.32 | 0.00 | NA | 0.01 | 0.70 | 0.68 | 0.72 |
| Self-control | 0.38 | 0.36 | 0.41 | 0.00 | NA | 0.01 | 0.62 | 0.59 | 0.64 |
| Consideration of future consequences | 0.24 | 0.17 | 0.29 | 0.02 | 0.00 | 0.07 | 0.74 | 0.71 | 0.77 |
| Risk-taking | 0.45 | 0.39 | 0.50 | 0.03 | 0.00 | 0.08 | 0.52 | 0.49 | 0.54 |
| Anxiety | 0.25 | 0.18 | 0.32 | 0.06 | 0.01 | 0.11 | 0.69 | 0.66 | 0.72 |
| Peer problems | 0.30 | 0.24 | 0.37 | 0.08 | 0.03 | 0.13 | 0.62 | 0.59 | 0.64 |
| Hyperactivity | 0.29 | 0.23 | 0.35 | 0.03 | 0.00 | 0.08 | 0.68 | 0.65 | 0.70 |
| Conduct problems | 0.19 | 0.12 | 0.26 | 0.05 | 0.00 | 0.11 | 0.76 | 0.73 | 0.78 |
| Depressive symptoms | 0.30 | 0.23 | 0.36 | 0.05 | 0.00 | 0.10 | 0.65 | 0.63 | 0.68 |
| Aggression | 0.33 | 0.31 | 0.35 | 0.00 | NA | 0.01 | 0.67 | 0.65 | 0.69 |
| Antisocial behavior | 0.28 | 0.26 | 0.31 | 0.00 | NA | 0.01 | 0.72 | 0.69 | 0.74 |
| Attention-deficit hyperactivity disorder | 0.43 | 0.40 | 0.45 | 0.00 | NA | 0.02 | 0.57 | 0.55 | 0.60 |
| Psychotic symptoms | 0.37 | 0.34 | 0.39 | 0.00 | NA | 0.03 | 0.63 | 0.61 | 0.66 |
| Subjective well-being | 0.28 | 0.21 | 0.34 | 0.03 | 0.00 | 0.08 | 0.69 | 0.66 | 0.72 |
| Factor 1 | 0.34 | 0.27 | 0.40 | 0.09 | 0.04 | 0.14 | 0.57 | 0.55 | 0.60 |
| Factor 2 | 0.47 | 0.44 | 0.49 | 0.00 | NA | 0.01 | 0.53 | 0.51 | 0.56 |

*Note:* Standardized estimates. A% = additive genetic variance component, C% = shared environmental variance component, E% = nonshared environmental variance component; lCI = 95% Confidence Intervals, lower bound, uCI = 95% Confidence Intervals, upper bound.

#### Supplementary Table 10 (ST10). Model fit indices, univariate twin models

|  | ep | minus2LL | *df* | AIC | BIC | CFI | TLI | RMSEA | diffLL | diffdf | *p* |
| --- | --- | --- | --- | --- | --- | --- | --- | --- | --- | --- | --- |
| Univariate twin models |  |  |  |  |  |  |  |  |  |  |  |
| Childhood maltreatment | 4 | 60697.60 | 22680 | 15337.60 | -151049.00 | 1.00 | 1.00 | 0.00 | 0.00 | 0.00 | 1.000 |
| IPV | 4 | 63942.31 | 22680 | 18582.31 | -147804.20 | 1.00 | 1.00 | 0.00 | 0.00 | 0.00 | 1.000 |
| Peer victimization | 4 | 63881.77 | 22680 | 18521.77 | -147864.80 | 1.00 | 1.00 | 0.00 | 0.86 | 0.00 | 1.000 |
| Neuroticism | 4 | 63541.56 | 22680 | 18181.56 | -148205.00 | 1.00 | 1.00 | 0.00 | 0.00 | 0.00 | 1.000 |
| Extraversion | 4 | 63388.16 | 22680 | 18028.16 | -148358.40 | 0.99 | 1.00 | 0.00 | 9.30 | 0.00 | 1.000 |
| Openness | 4 | 63584.11 | 22680 | 18224.11 | -148162.40 | 0.99 | 1.00 | 0.00 | 5.68 | 0.00 | 1.000 |
| Agreeableness | 4 | 64049.13 | 22680 | 18689.13 | -147697.40 | 0.87 | 1.00 | 0.00 | 48.37 | 0.00 | 1.000 |
| Conscientiousness | 4 | 63853.09 | 22680 | 18493.09 | -147893.50 | 0.92 | 1.00 | 0.00 | 47.93 | 0.00 | 1.000 |
| Self-control | 4 | 63477.07 | 22680 | 18117.07 | -148269.50 | 0.99 | 1.00 | 0.00 | 11.58 | 0.00 | 1.000 |
| Consideration of future consequences | 4 | 63933.38 | 22680 | 18573.38 | -147813.20 | 1.00 | 1.00 | 0.00 | 0.00 | 0.00 | 1.000 |
| Risk-taking | 4 | 62767.60 | 22680 | 17407.60 | -148979.00 | 1.00 | 1.00 | 0.00 | 0.00 | 0.00 | 1.000 |
| Anxiety | 4 | 63673.72 | 22680 | 18313.72 | -148072.80 | 1.00 | 1.00 | 0.00 | 0.00 | 0.00 | 1.000 |
| Peer problems | 4 | 63311.13 | 22680 | 17951.13 | -148435.40 | 1.00 | 1.00 | 0.00 | 0.00 | 0.00 | 1.000 |
| Hyperactivity | 4 | 63680.72 | 22680 | 18320.72 | -148065.80 | 1.00 | 1.00 | 0.00 | 0.00 | 0.00 | 1.000 |
| Conduct problems | 4 | 63948.85 | 22680 | 18588.85 | -147797.70 | 1.00 | 1.00 | 0.00 | 0.00 | 0.00 | 1.000 |
| Depressive symptoms | 4 | 63545.43 | 22680 | 18185.43 | -148201.10 | 1.00 | 1.00 | 0.00 | 0.00 | 0.00 | 1.000 |
| Aggression | 4 | 63701.81 | 22680 | 18341.81 | -148044.70 | 0.97 | 1.00 | 0.00 | 17.58 | 0.00 | 1.000 |
| Antisocial behavior | 4 | 63926.65 | 22680 | 18566.65 | -147819.90 | 0.95 | 1.00 | 0.00 | 24.42 | 0.00 | 1.000 |
| Psychotic symptoms | 4 | 63245.15 | 22680 | 17885.15 | -148501.40 | 1.00 | 1.00 | 0.00 | 3.87 | 0.00 | 1.000 |
| Attention-deficit hyperactivity disorder | 4 | 63522.23 | 22680 | 18162.23 | -148224.30 | 1.00 | 1.00 | 0.00 | 1.51 | 0.00 | 1.000 |
| Subjective well-being | 4 | 63734.71 | 22680 | 18374.71 | -148011.90 | 1.00 | 1.00 | 0.00 | 0.00 | 0.00 | 1.000 |
| Factor 1 | 4 | 60268.10 | 22680 | 14908.10 | -151478.50 | 1.00 | 1.00 | 0.00 | 0.00 | 0.00 | 1.00 |
| Factor 2 | 4 | 59624.97 | 22680 | 14264.97 | -152121.60 | 0.99 | 1.00 | 0.00 | 9.13 | 0.00 | 1.000 |

*Note*: ep = Estimated Parameter; minus2LL = Minus Two Log-Likelihood; *df* = Degrees of Freedom; AIC = Akaike Information Criterion; BIC = Bayesian Information Criterion; CFI = Comparative Fit Index; TLI = Tucker–Lewis Index; RMSEA = Root Mean Square Error of Approximation; diffLL = Difference in Log-Likelihood; diffdf = Difference in degrees of freedom; *p* = *p*-value. For each variable, fit statistics reflect comparison between the main ACE model and a saturated reference model, i.e., a fully parameterized, unconstrained model that freely estimates means, variances, and covariances without imposing structural constraints based on expected MZ and DZ twin correlations.

#### Supplementary Table 11 (ST11). Variance components and etiological correlations, separate multivariate twin models

|  | A% | lCI | uCI | C% | lCI | uCI | E% | lCI | uCI | *r_g_* | lCI | uCI | *r_c_* | lCI | uCI | *r_e_* | lCI | uCI |
| --- | --- | --- | --- | --- | --- | --- | --- | --- | --- | --- | --- | --- | --- | --- | --- | --- | --- | --- |
| Childhood maltreatment |  |  |  |  |  |  |  |  |  |  |  |  |  |  |  |  |  |  |
| Intimate partner violence victimization | 0.50 | 0.32 | 0.68 | 0.27 | 0.12 | 0.40 | 0.24 | 0.19 | 0.30 | 0.52 | 0.52 | 0.68 | 0.42 | 0.42 | 0.74 | 0.10 | 0.07 | 0.13 |
| Neuroticism | 0.48 | 0.30 | 0.67 | 0.20 | 0.05 | 0.34 | 0.32 | 0.26 | 0.39 | 0.34 | 0.22 | 0.46 | 0.48 | 0.48 | 1.00 | 0.14 | 0.11 | 0.17 |
| Extraversion | 0.30 | 0.03 | 0.58 | 0.48 | 0.28 | 0.70 | 0.22 | 0.11 | 0.32 | -0.11 | -0.22 | -0.01 | -0.91 | -1.00 | -0.91 | -0.06 | -0.09 | -0.03 |
| Openness | 0.48 | NA | NA | 0.35 | 0.19 | 0.35 | 0.17 | 0.04 | 0.36 | 0.17 | 0.06 | 0.29 | -0.97 | NA | -0.48 | 0.04 | 0.01 | 0.07 |
| Agreeableness | 0.49 | 0.19 | 0.65 | 0.04 | NA | 0.26 | 0.47 | 0.28 | 0.60 | -0.22 | -0.35 | -0.09 | -0.54 | -1.00 | 0.83 | -0.10 | -0.13 | -0.07 |
| Conscientiousness | 0.63 | 0.62 | 0.70 | 0.11 | 0.00 | 0.20 | 0.26 | 0.15 | 0.43 | -0.36 | -0.49 | -0.25 | 0.97 | NA | 1.00 | -0.09 | -0.11 | -0.06 |
| Self-control | 0.63 | 0.51 | 0.72 | 0.03 | 0.00 | 0.16 | 0.34 | 0.24 | 0.41 | 0.39 | 0.29 | 0.50 | 0.37 | -1.00 | 1.00 | 0.15 | 0.12 | 0.18 |
| Consideration of future consequences | 0.67 | 0.61 | 0.80 | 0.20 | 0.00 | 0.30 | 0.13 | 0.02 | 0.34 | 0.37 | 0.22 | 0.54 | -0.32 | -0.91 | NA | 0.03 | 0.01 | 0.06 |
| Risk-taking | 0.62 | 0.55 | 0.67 | 0.12 | 0.00 | 0.21 | 0.27 | 0.17 | 0.42 | 0.35 | 0.26 | 0.45 | -0.23 | -0.66 | -0.20 | 0.13 | 0.10 | 0.15 |
| Anxiety | 0.49 | 0.32 | 0.66 | 0.19 | 0.06 | 0.32 | 0.33 | 0.26 | 0.39 | 0.37 | 0.25 | 0.50 | 0.33 | 0.09 | 0.60 | 0.15 | 0.12 | 0.18 |
| Peer problems | 0.25 | NA | 0.42 | 0.51 | 0.38 | 0.63 | 0.24 | 0.18 | 0.29 | 0.20 | 0.07 | 0.31 | 0.72 | 0.54 | 0.90 | 0.11 | 0.09 | 0.14 |
| Hyperactivity | 0.55 | 0.38 | 0.73 | 0.18 | 0.04 | 0.32 | 0.27 | 0.21 | 0.34 | 0.41 | 0.28 | 0.54 | 0.46 | 0.46 | 1.00 | 0.12 | 0.09 | 0.15 |
| Conduct problems | 0.27 | 0.07 | 0.46 | 0.29 | 0.13 | 0.44 | 0.44 | 0.37 | 0.52 | 0.23 | 0.06 | 0.39 | 0.48 | 0.48 | 0.82 | 0.17 | 0.15 | 0.20 |
| Depressive symptoms | 0.31 | 0.17 | 0.46 | 0.32 | 0.20 | 0.43 | 0.38 | 0.32 | 0.43 | 0.30 | 0.17 | 0.43 | 0.75 | 0.75 | 1.00 | 0.21 | 0.18 | 0.23 |
| Aggression | 0.64 | 0.48 | 0.79 | 0.09 | 0.00 | 0.22 | 0.27 | 0.21 | 0.33 | 0.45 | 0.34 | 0.57 | 0.40 | NA | 1.00 | 0.12 | 0.09 | 0.14 |
| Antisocial behavior | 0.61 | 0.45 | 0.68 | 0.02 | NA | 0.12 | 0.37 | 0.25 | 0.46 | 0.44 | 0.32 | 0.57 | -0.20 | -1.00 | 1.00 | 0.15 | 0.12 | 0.18 |
| Attention-deficit hyperactivity disorder | 0.45 | 0.32 | 0.57 | 0.20 | 0.10 | 0.30 | 0.36 | 0.31 | 0.41 | 0.36 | 0.27 | 0.45 | 0.98 | 0.90 | 1.00 | 0.22 | 0.19 | 0.24 |
| Psychotic symptoms | 0.50 | 0.41 | 0.59 | 0.08 | NA | 0.15 | 0.42 | 0.39 | 0.46 | 0.64 | 0.55 | 0.73 | 0.53 | 0.53 | 1.00 | 0.35 | 0.32 | 0.37 |
| Subjective well-being | 0.52 | 0.33 | 0.69 | 0.12 | 0.00 | 0.26 | 0.36 | 0.30 | 0.43 | -0.39 | -0.52 | -0.26 | -0.28 | -1.00 | 1.00 | -0.16 | -0.19 | -0.13 |
| Intimate partner violence victimization |  |  |  |  |  |  |  |  |  |  |  |  |  |  |  |  |  |  |
| Neuroticism | 0.44 | 0.20 | 0.64 | 0.11 | 0.00 | 0.29 | 0.45 | 0.34 | 0.54 | 0.38 | 0.18 | 0.60 | 0.52 | 0.52 | 1.00 | 0.13 | 0.10 | 0.16 |
| Extraversion | 0.56 | 0.21 | 0.90 | 0.26 | 0.03 | 0.52 | 0.18 | 0.00 | 0.35 | -0.22 | -0.36 | -0.22 | -0.75 | -0.99 | -0.09 | -0.03 | -0.05 | 0.00 |
| Openness | 0.54 | 0.06 | 0.80 | 0.18 | 0.00 | 0.18 | 0.27 | 0.01 | 0.81 | 0.16 | 0.16 | 0.35 | -0.63 | -1.00 | -0.63 | 0.03 | 0.00 | 0.05 |
| Agreeableness | 0.57 | 0.17 | 0.81 | 0.03 | NA | 0.21 | 0.39 | 0.11 | 0.68 | -0.21 | -0.41 | -0.06 | 0.54 | -1.00 | 1.00 | -0.04 | -0.06 | -0.01 |
| Conscientiousness | 0.59 | 0.41 | 0.71 | 0.05 | 0.00 | 0.15 | 0.36 | 0.21 | 0.54 | -0.37 | -0.58 | -0.23 | 0.65 | -1.00 | 1.00 | -0.07 | -0.09 | -0.04 |
| Self-control | 0.49 | 0.29 | 0.63 | 0.04 | 0.00 | 0.18 | 0.47 | 0.35 | 0.57 | 0.35 | 0.22 | 0.51 | 1.00 | -1.00 | NA | 0.12 | 0.10 | 0.15 |
| Consideration of future consequences | 0.03 | NA | 0.47 | 0.33 | 0.00 | 0.45 | 0.64 | 0.46 | 0.77 | 0.02 | -0.24 | 0.02 | 0.74 | 0.01 | 0.94 | 0.09 | 0.06 | 0.11 |
| Risk-taking | 0.15 | NA | 0.46 | 0.33 | 0.10 | 0.50 | 0.52 | 0.40 | 0.60 | 0.07 | -0.02 | 0.07 | 0.80 | 0.26 | 0.95 | 0.11 | 0.08 | 0.14 |
| Anxiety | 0.34 | 0.17 | 0.52 | 0.27 | 0.15 | 0.39 | 0.39 | 0.32 | 0.46 | 0.38 | 0.23 | 0.53 | 1.00 | 0.97 | NA | 0.13 | 0.11 | 0.16 |
| Peer problems | 0.34 | 0.18 | 0.51 | 0.32 | 0.20 | 0.45 | 0.33 | 0.26 | 0.40 | 0.40 | 0.40 | 0.55 | 0.92 | 0.81 | 1.00 | 0.12 | 0.09 | 0.14 |
| Hyperactivity | 0.41 | 0.20 | 0.65 | 0.19 | 0.01 | 0.34 | 0.40 | 0.31 | 0.49 | 0.40 | 0.22 | 0.62 | 0.91 | 0.85 | 1.00 | 0.12 | 0.09 | 0.15 |
| Conduct problems | 0.29 | 0.12 | 0.48 | 0.26 | 0.13 | 0.39 | 0.45 | 0.38 | 0.53 | 0.41 | 0.21 | 0.64 | 1.00 | 0.89 | NA | 0.15 | 0.12 | 0.17 |
| Depressive symptoms | 0.40 | 0.24 | 0.58 | 0.20 | 0.06 | 0.32 | 0.40 | 0.33 | 0.47 | 0.50 | 0.35 | 0.72 | 0.91 | 0.32 | 1.00 | 0.15 | 0.12 | 0.17 |
| Aggression | 0.46 | 0.26 | 0.64 | 0.14 | 0.01 | 0.28 | 0.41 | 0.31 | 0.50 | 0.39 | 0.25 | 0.53 | 1.00 | 0.99 | NA | 0.11 | 0.08 | 0.13 |
| Antisocial behavior | 0.70 | 0.57 | 0.82 | 0.06 | 0.00 | 0.16 | 0.23 | 0.11 | 0.39 | 0.57 | 0.39 | 0.85 | -0.98 | NA | 1.00 | 0.05 | 0.03 | 0.08 |
| Attention-deficit hyperactivity disorder | 0.59 | 0.42 | 0.75 | 0.08 | 0.00 | 0.21 | 0.33 | 0.30 | 0.41 | 0.47 | 0.47 | 0.60 | 0.59 | -0.27 | 0.99 | 0.10 | 0.08 | 0.13 |
| Psychotic symptoms | 0.54 | 0.42 | 0.68 | 0.10 | 0.00 | 0.21 | 0.36 | 0.29 | 0.42 | 0.61 | 0.49 | 0.75 | 0.99 | NA | NA | 0.14 | 0.12 | 0.17 |
| Subjective well-being | 0.38 | 0.15 | 0.50 | 0.03 | NA | 0.21 | 0.59 | 0.41 | 0.68 | -0.38 | -0.61 | -0.38 | -0.16 | -1.00 | 1.00 | -0.18 | -0.20 | -0.15 |

*Note*: Standardized estimates. Each multivariate model comprised childhood maltreatment, intimate partner violence and the listed intermediary phenotype. A% = additive genetic covariation component, C% = shared environmental covariation component, E% = nonshared environmental covariation component for the intermediary phenotypes. Correlations between additive genetic, shared environmental and nonshared environmental components for each variable and childhood maltreatment/intimate partner violence (*r_g_*, *r_c_* and *r_e_*, respectively) are reported in separate rows for ease of representation., lCI = 95% Confidence Intervals, lower bound, uCI = 95% Confidence Intervals, upper bound, *r_g_* = genetic correlations, *r_c_* = shared environmental correlations, *r_e_* = nonshared environmental correlations.

#### Supplementary Table 12 (ST12). Model fit indices, multivariate twin models

|  | ep | minus2LL | *df* | AIC | BIC | CFI | TLI | RMSEA | diffLL | diffdf | *p* |
| --- | --- | --- | --- | --- | --- | --- | --- | --- | --- | --- | --- |
| Multivariate twin models |  |  |  |  |  |  |  |  |  |  |  |
| CM, IPV | 11 | 123450.80 | 45357 | 32736.82 | -300014.30 | 1.00 | 1.00 | 0.00 | 0.02 | 2.00 | 0.992 |
| CM, IPV, Neuroticism | 21 | 185354.90 | 68031 | 49292.91 | -449800.70 | 1.00 | 1.00 | 0.00 | 1.13 | 6.00 | 0.980 |
| CM, IPV, Extraversion | 21 | 186389.70 | 68031 | 50327.71 | -448765.90 | 1.00 | 0.99 | 0.01 | 18.19 | 6.00 | 0.006 |
| CM, IPV, Openness | 21 | 186948.90 | 68031 | 50886.88 | -448206.80 | 1.00 | 1.00 | 0.01 | 9.62 | 6.00 | 0.142 |
| CM, IPV, Agreeableness | 21 | 187126.10 | 68031 | 51064.07 | -448029.60 | 0.99 | 0.97 | 0.03 | 51.00 | 6.00 | < 0.001 |
| CM, IPV, Conscientiousness | 21 | 186667.40 | 68031 | 50605.44 | -448488.20 | 0.99 | 0.98 | 0.03 | 49.47 | 6.00 | < 0.001 |
| CM, IPV, Self-control | 21 | 185505.10 | 68031 | 49443.15 | -449650.50 | 1.00 | 1.00 | 0.01 | 12.63 | 6.00 | 0.049 |
| CM, IPV, Consideration of future consequences | 21 | 187043.00 | 68031 | 50980.97 | -448112.70 | 1.00 | 1.00 | 0.00 | 0.63 | 6.00 | 0.996 |
| CM, IPV, Risk-taking | 21 | 185366.50 | 68031 | 49304.46 | -449789.20 | 1.00 | 1.00 | 0.00 | 0.56 | 6.00 | 0.997 |
| CM, IPV, Anxiety | 21 | 184972.80 | 68031 | 48910.81 | -450182.80 | 1.00 | 1.00 | 0.00 | 1.71 | 6.00 | 0.945 |
| CM, IPV, Peer problems | 21 | 184708.70 | 68031 | 48646.67 | -450447.00 | 1.00 | 1.00 | 0.00 | 3.27 | 6.00 | 0.775 |
| CM, IPV, Hyperactivity | 21 | 185390.00 | 68031 | 49328.01 | -449765.60 | 1.00 | 1.00 | 0.00 | 0.81 | 6.00 | 0.992 |
| CM, IPV, Conduct problems | 21 | 185411.50 | 68031 | 49349.46 | -449744.20 | 1.00 | 1.00 | 0.00 | 1.33 | 6.00 | 0.970 |
| CM, IPV, Depressive symptoms | 21 | 184335.20 | 68031 | 48273.25 | -450820.40 | 1.00 | 1.00 | 0.00 | 0.43 | 6.00 | 0.999 |
| CM, IPV, Aggression | 21 | 185634.30 | 68031 | 49572.26 | -449521.40 | 1.00 | 0.99 | 0.02 | 24.72 | 6.00 | < 0.001 |
| CM, IPV, Antisocial behavior | 21 | 186190.70 | 68031 | 50128.69 | -448965.00 | 1.00 | 0.99 | 0.02 | 22.90 | 6.00 | 0.001 |
| CM, IPV, Psychotic symptoms | 21 | 184346.80 | 68031 | 48284.84 | -450808.80 | 1.00 | 1.00 | 0.01 | 9.98 | 6.00 | 0.125 |
| CM, IPV, Attention-deficit hyperactivity disorder | 21 | 181799.60 | 68031 | 45737.60 | -453356.00 | 1.00 | 1.00 | 0.00 | 7.13 | 6.00 | 0.309 |
| CM, IPV, Subjective well-being | 21 | 185395.80 | 68031 | 49333.83 | -449759.80 | 1.00 | 1.00 | 0.00 | 0.33 | 6.00 | 0.999 |
| CM, IPV, Factor 1 | 21 | 179795.50 | 68031 | 43733.54 | -455360.10 | 1.00 | 1.00 | 0.00 | 0.34 | 6.00 | 0.999 |
| CM, IPV, Factor 2 | 21 | 179707.00 | 68031 | 43645.02 | -455448.60 | 1.00 | 1.00 | 0.01 | 14.68 | 6.00 | 0.023 |

*Note*: CM = Childhood maltreatment, IPV = Intimate partner violence, ep = Estimated Parameter; minus2LL = Minus Two Log-Likelihood; *df* = Degrees of Freedom; AIC = Akaike Information Criterion; BIC = Bayesian Information Criterion; CFI = Comparative Fit Index; TLI = Tucker–Lewis Index; RMSEA = Root Mean Square Error of Approximation; diffLL = Difference in Log-Likelihood; diffdf = Difference in degrees of freedom; *p* = *p*-value. For each variable, fit statistics reflect comparison between the main ACE model and a saturated reference model, i.e., a fully parameterized, unconstrained model that freely estimates means, variances, and covariances without imposing structural constraints based on expected MZ and DZ twin correlations. Significant model fit differences (p < 0.001) for agreeableness, conscientiousness, and aggression, indicate that the multivariate ACE model deviated from the saturated model. This likely reflects instability in the shared environmental (C) components and their cross-variable correlations (see estimates and boundary values in Table 11), rather than genuine misspecification of the ACE structure.

## Supplementary Materials 6. Linkage Disequilibrium score regression (LDSC)

### Methods

For linkage disequilibrium score regression (LDSC) analyses ^48,49^, we used recent genome wide association study (GWAS) summary statistics based on individuals with European genetic ancestry. For consistency, we used the same GWAS summary statistics used to generate PGSs in TEDS, except for the summary statistics of child (vs. adult) aggression ^35^. We additionally used four GWAS summary statistics that lacked corresponding PGSs in TEDS—namely emotional, physical, and sexual IPV victimization (nealelab.is/uk-biobank/) and antisocial behavior ^50^. We used summary statistics with partly overlapping samples as the sampling covariance matrices in LDSC are adjusted for sample overlap. Before analysis, we performed standard quality control of summary statistics ^51^, which included restricting variants to those located on autosomal chromosomes, retaining common variants at minor allele frequency > 0.1, excluding strand ambiguous single nucleotide polymorphisms (SNPs) and retaining well-imputed variants based on INFO > 0.8.

We conducted LDSC ^52^ with the R package GenomicSEM ^53^ to estimate the extent of the SNP-heritability (*h^2^_SNP_*) and genetic correlations (*r_g_*) between each victimization experience (maltreatment and IPV victimization) and the personality and mental health phenotypes that showed significant associations with such experiences in GEE analyses. We applied FDR correction ^54^ to account for multiple testing. LDSC relies on the fact that, due to Linkage Disequilibrium (LD), alleles are non-randomly associated based on their location on a chromosome. By examining how strongly SNPs are in LD with each other relative to their effects on a phenotype, LDSC estimates the overall SNP heritability (*h^2^_SNP_*) of a trait. By extension, bivariate LDSC estimates the genetic correlation (*r_g_* ) between two phenotypes ^48^.

### Results

LDSC indicated small *h^2^_SNP_* estimates (see [Supplementary Table 13](#_Supplementary_Table_11._1)), meaning that common genetic variants explained a modest proportion of variance across victimization experiences, personality, and mental health phenotypes. All estimates were statistically significant except for conscientiousness. Externalizing traits showed slightly higher *h^2^_SNP_*, and schizophrenia showed the highest estimate (*h^2^_SNP_* = 0.20). Heritability estimates were generally smaller than those reported in the original studies. This discrepancy could be attributed to differences in samples (e.g., our GWAS summary statistics were obtained from samples excluding 23andMe participants), differences in quality control of summary statistics (e.g., we excluded strand-ambiguous SNPs), and differences in heritability estimation methods (e.g. Heritability Estimation from Summary Statistics, BOLT-LMM, or SBayesS, rather than LDSC).

Genetic correlations are reported in [Supplementary Table 14](#_Supplementary_Table_13._1). Maltreatment showed the strongest genetic correlations with physical, emotional, and sexual IPV victimization. Among the personality and mental health phenotypes, maltreatment showed the strongest genetic correlations with aggression, depressive symptoms, and ADHD. Emotional, physical, and sexual IPV victimization were strongly genetically correlated. Among the personality and mental health phenotypes, depressive symptoms and anxiety showed the strongest genetic correlations with emotional and sexual IPV victimization. Depressive symptoms and ADHD showed the strongest genetic correlations with physical IPV victimization. Conscientiousness was not genetically associated with other variables, which was expected given its non-significant *h^2^_SNP_*. We also observed non-significant genetic correlations between sexual IPV and aggression, antisocial behaviour, and subjective wellbeing, as well as between risk-taking and anxiety and low subjective well-being.

#### Supplementary Table 13 (ST13). SNP heritability estimates, Linkage Disequilibrium score regression

| Summary statistics | *h²* | *h²* SE | h² Z | Mean Chi² | Lambda GC | Intercept | Intercept SE | Ratio | Ratio SE |
| --- | --- | --- | --- | --- | --- | --- | --- | --- | --- |
| Childhood maltreatment | 0.04 | 0.00 | 12.50 | 1.28 | 1.23 | 1.01 | 0.01 | 0.03 | 0.04 |
| IPV emotional | 0.02 | 0.00 | 7.27 | 1.10 | 1.09 | 1.00 | 0.01 | -0.03 | 0.09 |
| IPV physical | 0.01 | 0.00 | 5.07 | 1.07 | 1.08 | 1.01 | 0.01 | 0.13 | 0.12 |
| IPV sexual | 0.01 | 0.00 | 3.03 | 1.04 | 1.04 | 1.00 | 0.01 | 0.01 | 0.25 |
| Neuroticism | 0.06 | 0.00 | 19.60 | 1.86 | 1.63 | 1.03 | 0.02 | 0.04 | 0.02 |
| Conscientiousness | 0.03 | 0.03 | 0.99 | 1.02 | 1.02 | 1.01 | 0.01 | 0.20 | 0.54 |
| Risk-taking | 0.06 | 0.00 | 16.70 | 1.73 | 1.52 | 1.00 | 0.02 | 0.00 | 0.02 |
| Anxiety | 0.06 | 0.01 | 9.42 | 1.16 | 1.14 | 0.99 | 0.01 | -0.08 | 0.06 |
| Aggression | 0.02 | 0.00 | 6.32 | 1.11 | 1.10 | 1.01 | 0.01 | 0.12 | 0.09 |
| Antisocial behavior | 0.04 | 0.01 | 4.51 | 1.09 | 1.09 | 1.02 | 0.01 | 0.22 | 0.11 |
| Externalizing | 0.03 | 0.00 | 18.70 | 2.39 | 1.92 | 0.99 | 0.03 | 0.00 | 0.02 |
| ADHD | 0.08 | 0.01 | 14.40 | 1.45 | 1.34 | 1.02 | 0.01 | 0.05 | 0.03 |
| Schizophrenia | 0.20 | 0.01 | 20.60 | 2.10 | 1.80 | 1.11 | 0.02 | 0.10 | 0.02 |
| Subjective well-being | 0.01 | 0.00 | 8.47 | 1.15 | 1.12 | 1.00 | 0.01 | 0.03 | 0.07 |

*Note*: IPV = Intimate partner violence victimization; ADHD = Attention-deficit hyperactivity disorder; *h*² = SNP heritability estimate, *h*² SE = standard error of SNP heritability; *h*² Z = Z-score for SNP heritability; Mean Chi² = mean chi-square statistic; Lambda GC = genomic control inflation factor; Intercept = LD score regression intercept; Intercept SE = standard error of intercept; Ratio = proportion of inflation due to confounding; Ratio SE = standard error of the ratio.

#### Supplementary Table 14 (ST14). Genetic correlations, Linkage Disequilibrium score regression

|  |  | 1 | 2 | 3 | 4 | 5 | 6 | 7 | 8 | 9 | 10 | 11 | 12 | 13 | 14 | 15 |
| --- | --- | --- | --- | --- | --- | --- | --- | --- | --- | --- | --- | --- | --- | --- | --- | --- |
| 1 | Childhood maltreatment | **1.00** |  |  |  |  |  |  |  |  |  |  |  |  |  |  |
| 2 | IPV emotional | **0.73** | **1.00** |  |  |  |  |  |  |  |  |  |  |  |  |  |
| 3 | IPV physical | **0.82** | **0.78** | **1.00** |  |  |  |  |  |  |  |  |  |  |  |  |
| 4 | IPV sexual | **0.60** | **0.79** | **0.86** | **1.00** |  |  |  |  |  |  |  |  |  |  |  |
| 5 | Neuroticism | **0.40** | **0.41** | **0.45** | **0.27** | **1.00** |  |  |  |  |  |  |  |  |  |  |
| 6 | Conscientiousness | -0.27 | 0.15 | 0.03 | 0.30 | -0.03 | 1.00 |  |  |  |  |  |  |  |  |  |
| 7 | Risk-taking | **0.29** | **0.30** | **0.44** | **0.38** | **0.01** | **-0.23** | **1.00** |  |  |  |  |  |  |  |  |
| 8 | Anxiety | **0.49** | **0.53** | **0.59** | **0.46** | **0.67** | **-0.29** | 0.14 | 1.00 |  |  |  |  |  |  |  |
| 9 | Depression | **0.54** | **0.57** | **0.66** | **0.50** | **0.69** | **-0.18** | **0.29** | **0.80** | **1.00** |  |  |  |  |  |  |
| 10 | Aggression | **0.59** | **0.37** | **0.59** | 0.18 | **0.35** | **-0.50** | **0.38** | **0.46** | **0.00** | **1.00** |  |  |  |  |  |
| 11 | Antisocial behavior | **0.51** | **0.40** | **0.50** | 0.16 | **0.28** | **-0.59** | **0.48** | **0.47** | **0.00** | **0.94** | **1.00** |  |  |  |  |
| 12 | Externalizing | **0.46** | **0.46** | **0.61** | **0.42** | **0.12** | **-0.33** | **0.86** | **0.27** | **0.00** | **0.63** | **0.72** | **1.00** |  |  |  |
| 13 | ADHD | **0.54** | **0.48** | **0.62** | **0.33** | **0.29** | **-0.30** | **0.43** | **0.42** | **0.00** | **0.91** | **0.92** | **0.66** | **1.00** |  |  |
| 14 | Schizophrenia | **0.40** | **0.28** | **0.36** | **0.35** | **0.21** | **-0.17** | **0.14** | **0.35** | **0.00** | **0.23** | **0.17** | **0.22** | **0.23** | **1.00** |  |
| 15 | Subjective well-being | **-0.50** | **-0.38** | **-0.54** | -0.34 | **-0.65** | **0.21** | -0.11 | **-0.60** | **0.00** | **-0.43** | **-0.37** | **-0.23** | **-0.33** | **-0.32** | **1.00** |

*Note:* IPV = Intimate partner violence victimization; ADHD = Attention-deficit hyperactivity disorder. Statistically significant estimates (*p__adj_* < 0.01) are highlighted in bold font.

## Supplementary Materials 7. Polygenic score regression

### Methods

We conducted a multivariate regression analysis using the lm function ^55^ to examine the associations between the PGSs for childhood maltreatment and for our candidate mediators, on one hand, and IPV victimization, on the other. We excluded candidate mediators showing non-significant associations with IPV (i.e., openness) and those showing non-significant associations with their corresponding PGS (i.e., conscientiousness and psychotic experiences) in GEE. We used PGSs created by the TEDS team using LDPred ^56^. When TEDS had been included in the discovery genome wide association study (GWAS) sample, PGS were created from summary statistics generated without TEDS data, preventing sample overlap. When generating PGSs, a prior on the fraction of causal markers of one was applied, assuming an infinitesimal model where all genetic markers are involved in trait development. See ^57^ for details of genotyping and quality control. We adjusted for potential confounders including batch effects, population structure (first ten principal components), sex, and age, and we applied Bonferroni correction.

### Results

Results are reported in [Supplementary Table 15](#_Supplementary_Table_14.). The PGSs for externalizing behaviors, depression, and ADHD were significantly associated with IPV victimization—suggesting that these genetic predispositions may also increase risk for IPV. The PGSs for maltreatment and aggression were nominally significant but did not survive Bonferroni correction. The model was statistically significant, F(22, 6498) = 8.93, *p* < .001, explaining 3% of the variance in IPV victimization (*R²* = 0. 029, *R²_adj_* = 0.026). Age was a significant covariate.

#### Supplementary Table 15 (ST15). Estimates, polygenic score regression of intimate partner violence victimization on polygenic scores

|  | Estimate | lCI | uCI | *t* | *p* | *p__adj_* |
| --- | --- | --- | --- | --- | --- | --- |
| Polygenic score |  |  |  |  |  |  |
| Childhood maltreatment | -0.28 | -0.49 | -0.07 | -2.63 | 0.009 | 0.078 |
| Neuroticism | -0.02 | -0.15 | 0.11 | -0.30 | 0.763 | 1.000 |
| Risk tolerance | -0.07 | -0.29 | 0.15 | -0.65 | 0.516 | 1.000 |
| Externalizing | 0.34 | 0.17 | 0.51 | 3.88 | < 0.001 | 0.001 |
| Aggression | 0.71 | 0.16 | 1.25 | 2.55 | 0.011 | 0.096 |
| Depression | 0.16 | 0.08 | 0.24 | 3.90 | < 0.001 | 0.001 |
| Anxiety | -0.04 | -0.11 | 0.03 | -1.20 | 0.230 | 1.000 |
| ADHD | 0.10 | 0.05 | 0.15 | 4.06 | < 0.001 | 0.000 |
| Subjective well-being | -0.08 | -0.32 | 0.16 | -0.67 | 0.502 | 1.000 |
| Covariate |  |  |  |  |  |  |
| Batch number | 0.00 | -0.01 | 0.00 | -1.13 | 0.259 | 1.000 |
| Principal component 1 | 1.09 | -1.43 | 3.62 | 0.85 | 0.397 | 1.000 |
| Principal component 2 | -3.29 | -5.76 | -0.81 | -2.60 | 0.009 | 0.121 |
| Principal component 3 | 1.45 | -1.02 | 3.93 | 1.15 | 0.251 | 1.000 |
| Principal component 4 | 1.71 | -0.73 | 4.15 | 1.37 | 0.170 | 1.000 |
| Principal component 5 | 1.29 | -1.18 | 3.77 | 1.02 | 0.306 | 1.000 |
| Principal component 6 | -0.75 | -3.23 | 1.72 | -0.60 | 0.550 | 1.000 |
| Principal component 7 | -0.77 | -3.27 | 1.73 | -0.60 | 0.546 | 1.000 |
| Principal component 8 | 0.20 | -2.25 | 2.66 | 0.16 | 0.870 | 1.000 |
| Principal component 9 | -1.82 | -4.30 | 0.66 | -1.44 | 0.150 | 1.000 |
| Principal component 10 | 3.66 | 1.21 | 6.11 | 2.93 | 0.003 | 0.045 |
| Sex | -0.01 | -0.06 | 0.04 | -0.23 | 0.818 | 1.000 |
| Age | 0.05 | 0.02 | 0.07 | 3.49 | < 0.001 | 0.006 |

*Note:* Standardized estimates. lCI = 95% confidence interval, lower bound; uCI = 95% confidence interval, upper bound; *t* = t-statistic; *p__adj_* = Bonferroni-adjusted *p*-value.

## Supplementary Materials 8. Mendelian Randomization Direction of Causation

### Methods

We conducted Mendelian Randomization Direction of Causation (MR-DoC^58^; Supplementary Figure SF1) using the R package OpenMx ^59^. Standard MR ^60,61^ typically rely on a limited number of genome-wide significant SNPs associated with an exposure (e.g., maltreatment) as instrumental variables for estimating its causal effect on an outcome (e.g. IPV victimization). MR assumes that instrumental variables are associated with the exposure, independent of confounders, and not independently associated with the outcome. However, individual SNPs have weaker associations with exposures, therefore introducing risk for weak instrument bias, as well as additional bias from horizontal, or unmediated pleiotropy (i.e. genetic variants directly affecting outcomes as well as exposures), thereby affecting the validity of causal effect estimates. In contrast, MR-DoC uses a PGS which has a stronger association with the exposure than individual SNPs, increasing instrument strength. It explicitly models horizontal pleiotropy by including direct paths from the PGS to all variables in the model, addressing violations of MR’s exclusion restriction assumption (the assumption that genetic instruments affect outcomes only through the exposure of interest). By partitioning genetic effects into vertical and horizontal pleiotropic effects and accounting for these when estimating causal effects, MR-DoC produces less biased estimates. A second causal inference method, DoC ^62,63^, estimates latent genetic and environmental variance components of an exposure and uses them as instrumental variables to predict an outcome. It assumes that variance components influencing exposure and outcome are distinct, which is often violated due to most behavioral traits sharing genetic and environmental components. MR-DoC improves on DoC twin models by not requiring different patterns of genetic and environmental influences on exposure and outcome to estimate unidirectional causal effects. By explicitly modeling genetic and shared environmental correlations between the variables in the model, it further minimises etiological confounding of causal effects.

#### Supplementary Figure SF1.

Path diagram for a Mendelian Randomisation-Direction of Causation (MR-DoC) model testing causal relationships between an Exposure (f1) and Outcome (f2) via a Mediator (m1)^64^. The model combines the Direction of Causation (DoC) twin model (red dashed box) with Mendelian Randomisation (MR; blue dashed box) to establish causal effects whilst accounting for potential confounding. Consistent with the conventions of structural equation modelling, rectangles denote observed variables; circles denote latent variables.

**DoC component:** The Direction of Causation (DoC) model decomposes the variance in the exposure, outcome, and mediator into three variance components: additive genetic influences (A), shared environmental influences (C), and nonshared environmental influences (E). A represents the sum of effects from multiple genetic variants influencing the trait, C represents environmental factors that are common to both twins in a pair and make them similar to one another, and E represents environmental factors that are unique to each individual twin and make them different from one another. The standardised path coefficients representing the effects of A, C, and E influences on the exposure are denoted as af1, cf1, ef1, while am1, cm1, and em1 denote influences on the mediator and af2, cf2, and ef2 denote influences on the outcome. The model also estimates unidirectional causality (c’) while controlling for genetic correlations (*r_a_*) and shared environmental correlations (*r_c_*). i.e., the extent to which A and C influences on the exposure, mediator, and outcome overlap, with a value of 0 indicating no shared influences and a value of 1.0 indicating complete overlap (i.e., the same genetic/environmental factors influencing both exposure and outcome). Nonshared environmental correlations (*r_e_*) are not estimated to ensure model identification.

**MR component:** The MR component incorporates the polygenic score for the exposure variable (PGS_exp_) as a statistical genetic instrument. PGS_exp_ indexes the weighted sum of all genetic variants across the genome that are associated with the exposure from an independent genome-wide association study, modelled as a latent factor (GP_exp_) for structural equation modelling. The path from the genetic instrument to the exposure (g₁, instrumental path), represents how well the genetic instrument predicts the exposure. The direct paths from the genetic instrument to the outcome (g₂) and the mediator (g_3_) independent of the exposure, called pleiotropic paths, control for horizontal pleiotropy (the phenomenon where genetic variants influence the outcome through pathways other than the exposure) which violates the assumptions of a statistical instrument. By modelling these paths, the MR-DoC derives a less biased estimate of causal effects.

**Mediation pathways:** Lastly, the mediation pathways test whether the causal effect of the exposure on the outcome operates through an intermediary mechanism (the Mediator), after accounting for all genetic and environmental confounding captured in the model. Mediation coefficients represent: *a* = effect of the exposure on the mediator; *b* = effect of the mediator on the outcome, controlling for the exposure; *c'* = direct effect of the exposure on the outcome controlling for the effect transmitted through the mediator. Additional mediation coefficients not illustrated in the Figure include: *ab* = indirect effect, representing the portion of the total effect of the exposure on the outcome that operates through the mediator (*a x b*); *c* = total effect, representing the overall effect of the exposure on the outcome (*c' + ab*). The proportion of the total causal effect that is mediated is calculated as: *ab/c,* expressed as a percentage.

### Results

#### Supplementary Table S16 (ST16). Estimates, genetically informed simple mediation (MR-DoC) models with freely estimated shared environmental parameters

|  | *a* | lCI | uCI | *b* | lCI | uCI | *c'* | lCI | uCI | *ab* | lCI | uCI | Pleiotropic path | lCI | uCI |
| --- | --- | --- | --- | --- | --- | --- | --- | --- | --- | --- | --- | --- | --- | --- | --- |
| Neuroticism | **0.18** | **0.15** | **0.22** | **0.13** | **0.10** | **0.16** | **0.12** | **0.08** | **0.16** | **0.02** | **0.02** | **0.03** | **0.04** | **0.02** | **0.05** |
| Extraversion | -0.07 | -0.11 | -0.04 | -0.02 | -0.05 | 0.01 | **0.14** | **0.10** | **0.18** | 0.00 | 0.00 | 0.00 | -0.03 | -0.04 | -0.01 |
| Agreeableness | **-0.15** | **-0.19** | **-0.11** | -0.03 | -0.05 | 0.00 | **0.14** | **0.10** | **0.18** | 0.00 | 0.00 | 0.01 | -0.01 | -0.02 | 0.01 |
| Conscientiousness | **-0.12** | **-0.15** | **-0.08** | **-0.06** | **-0.09** | **-0.04** | **0.14** | **0.10** | **0.18** | **0.01** | **0.01** | **0.01** | -0.02 | -0.04 | -0.01 |
| Self-control | **0.19** | **0.15** | **0.22** | **0.12** | **0.09** | **0.15** | **0.12** | **0.08** | **0.16** | **0.02** | **0.02** | **0.03** | 0.02 | 0.00 | 0.03 |
| Consideration of future consequences | 0.05 | 0.01 | 0.09 | **0.09** | **0.06** | **0.11** | **0.14** | **0.10** | **0.18** | 0.00 | 0.00 | 0.01 | 0.00 | -0.02 | 0.01 |
| Risk-taking | **0.14** | **0.11** | **0.18** | **0.12** | **0.09** | **0.15** | **0.13** | **0.09** | **0.17** | **0.02** | **0.01** | **0.02** | 0.00 | -0.02 | 0.02 |
| Anxiety | **0.19** | **0.15** | **0.23** | **0.13** | **0.10** | **0.16** | **0.12** | **0.08** | **0.15** | **0.03** | **0.02** | **0.03** | 0.03 | 0.01 | 0.04 |
| Peer problems | **0.14** | **0.11** | **0.18** | **0.12** | **0.09** | **0.15** | **0.13** | **0.09** | **0.16** | **0.02** | **0.01** | **0.02** | 0.05 | 0.03 | 0.07 |
| Hyperactivity | **0.16** | **0.12** | **0.20** | **0.11** | **0.09** | **0.14** | **0.12** | **0.09** | **0.16** | **0.02** | **0.01** | **0.02** | 0.03 | 0.02 | 0.05 |
| Conduct problems | **0.24** | **0.20** | **0.28** | **0.13** | **0.11** | **0.16** | **0.11** | **0.07** | **0.15** | **0.03** | **0.02** | **0.03** | 0.02 | 0.01 | 0.04 |
| Depressive symptoms | **0.27** | **0.24** | **0.31** | **0.14** | **0.11** | **0.17** | **0.10** | **0.06** | **0.14** | **0.04** | **0.03** | **0.04** | 0.03 | 0.01 | 0.04 |
| Aggression | **0.15** | **0.12** | **0.19** | **0.10** | **0.08** | **0.13** | **0.13** | **0.09** | **0.17** | **0.02** | **0.01** | **0.02** | **0.05** | **0.03** | **0.06** |
| Antisocial behavior | **0.20** | **0.16** | **0.24** | 0.04 | 0.01 | 0.07 | **0.14** | **0.10** | **0.18** | **0.01** | **0.01** | **0.01** | 0.03 | 0.01 | 0.04 |
| Attention-deficit hyperactivity disorder | **0.26** | **0.23** | **0.30** | **0.10** | **0.07** | **0.13** | **0.12** | **0.08** | **0.16** | **0.03** | **0.02** | **0.03** | 0.03 | 0.01 | 0.04 |
| Psychotic experiences | **0.45** | **0.41** | **0.48** | **0.13** | **0.10** | **0.16** | 0.08 | 0.04 | 0.12 | **0.06** | **0.05** | **0.07** | 0.01 | 0.00 | 0.03 |
| Subjective well-being | **-0.21** | **-0.25** | **-0.17** | **-0.17** | **-0.20** | **-0.14** | **0.11** | **0.07** | **0.15** | **0.04** | **0.03** | **0.04** | 0.00 | -0.01 | 0.02 |

*Note*. Standardized estimates. *a*=effect of childhood maltreatment on the candidate mediator; *b*=effect of the candidate mediator on intimate partner violence (IPV) victimization; *c’*=direct effect of maltreatment on IPV victimization controlling for the mediator; *ab*=indirect effect of maltreatment on IPV victimization via the mediator; lCI = 95% confidence intervals, lower bound; uCI = 95% confidence intervals, upper bound. In each model, the instrumental path from the PGS of childhood maltreatment to childhood maltreatment measured in TEDS was 0.09 (0.07, 0.11), the pleiotropic path from the PGS of childhood maltreatment to IPV victimization was 0.06 (0.04, 0.07). The total effect of maltreatment on IPV victimization was *c*=0.14 (0.13, 0.15). Statistically significant estimates (*p__adj_* < 0.003) are highlighted in bold font.

#### Supplementary Table 17 (ST17). Estimates, phenotypic simple mediation (SEM) models

| Mediator | *a* | lCI | uCI | *b* | lCI | uCI | *c'* | lCI | uCI | *ab* | lCI | uCI |
| --- | --- | --- | --- | --- | --- | --- | --- | --- | --- | --- | --- | --- |
| Neuroticism | 0.24 | 0.22 | 0.24 | 0.17 | 0.15 | 0.18 | 0.20 | 0.18 | 0.21 | 0.04 | 0.04 | 0.04 |
| Extraversion | 0.13 | 0.12 | 0.15 | 0.06 | 0.05 | 0.09 | 0.23 | 0.21 | 0.24 | 0.01 | 0.01 | 0.01 |
| Agreeableness | 0.11 | 0.10 | 0.13 | 0.05 | 0.03 | 0.07 | 0.23 | 0.21 | 0.24 | 0.01 | 0.00 | 0.01 |
| Conscientiousness | 0.13 | 0.12 | 0.14 | 0.10 | 0.07 | 0.11 | 0.22 | 0.21 | 0.23 | 0.01 | 0.01 | 0.02 |
| Self-control | 0.21 | 0.20 | 0.21 | 0.14 | 0.12 | 0.15 | 0.21 | 0.19 | 0.22 | 0.03 | 0.03 | 0.03 |
| Consideration of future consequences | 0.07 | 0.05 | 0.08 | 0.08 | 0.06 | 0.10 | 0.23 | 0.21 | 0.24 | 0.01 | 0.00 | 0.01 |
| Risk-taking | 0.16 | 0.13 | 0.17 | 0.10 | 0.07 | 0.12 | 0.22 | 0.20 | 0.23 | 0.02 | 0.01 | 0.02 |
| Anxiety | 0.23 | 0.23 | 0.24 | 0.19 | 0.17 | 0.20 | 0.19 | 0.18 | 0.20 | 0.04 | 0.04 | 0.05 |
| Peer problems | 0.22 | 0.19 | 0.23 | 0.20 | 0.19 | 0.22 | 0.19 | 0.18 | 0.20 | 0.04 | 0.04 | 0.05 |
| Hyperactivity | 0.23 | 0.22 | 0.23 | 0.18 | 0.16 | 0.19 | 0.19 | 0.18 | 0.21 | 0.04 | 0.04 | 0.05 |
| Conduct problems | 0.22 | 0.20 | 0.24 | 0.21 | 0.20 | 0.22 | 0.19 | 0.17 | 0.20 | 0.05 | 0.04 | 0.05 |
| Depressive symptoms | 0.27 | 0.26 | 0.28 | 0.21 | 0.18 | 0.23 | 0.18 | 0.16 | 0.19 | 0.06 | 0.05 | 0.06 |
| Aggression | 0.22 | 0.20 | 0.24 | 0.16 | 0.14 | 0.17 | 0.20 | 0.18 | 0.21 | 0.03 | 0.03 | 0.04 |
| Antisocial behavior | 0.19 | 0.17 | 0.21 | 0.08 | 0.07 | 0.09 | 0.22 | 0.20 | 0.23 | 0.02 | 0.01 | 0.02 |
| Attention-deficit hyperactivity disorder | 0.26 | 0.25 | 0.27 | 0.16 | 0.13 | 0.16 | 0.19 | 0.18 | 0.21 | 0.04 | 0.04 | 0.05 |
| Psychotic experiences | 0.40 | 0.38 | 0.43 | 0.20 | 0.18 | 0.21 | 0.16 | 0.14 | 0.17 | 0.08 | 0.07 | 0.09 |
| Subjective well-being | 0.21 | 0.19 | 0.23 | 0.19 | 0.16 | 0.21 | 0.20 | 0.18 | 0.20 | 0.04 | 0.03 | 0.05 |

*Note*. Standardized estimates. *a*=effect of childhood maltreatment on the candidate mediator; *b*=effect of the candidate mediator on intimate partner violence (IPV) victimization; *c’*=direct effect of maltreatment on IPV victimization controlling for the mediator; *ab*=indirect effect of maltreatment on IPV victimization via the mediator; lCI = 95% confidence intervals, lower bound; uCI = 95% confidence intervals, upper bound. In each model, the total effect of maltreatment on IPV victimization was *c*=0.24 (0.22, 0.25). All estimates are statistically significant (*p__adj_* < 0.003).

#### Supplementary Table 18 (ST18). Estimates, phenotypic parallel mediation (SEM) models (Models 1 and 2)

| Mediator | *a* | lCI | uCI | *b* | lCI | uCI | *ab* | lCI | uCI |
| --- | --- | --- | --- | --- | --- | --- | --- | --- | --- |
| Model 1 |  |  |  |  |  |  |  |  |  |
| Neuroticism | **0.24** | **0.22** | **0.25** | 0.02 | -0.01 | 0.05 | 0.01 | 0.00 | 0.01 |
| Extraversion | **0.13** | **0.11** | **0.15** | -0.03 | -0.06 | -0.01 | 0.00 | -0.01 | 0.00 |
| Agreeableness | **0.11** | **0.09** | **0.13** | -0.05 | -0.07 | -0.02 | -0.01 | -0.01 | 0.00 |
| Conscientiousness | **0.13** | **0.11** | **0.16** | 0.01 | -0.02 | 0.04 | 0.00 | 0.00 | 0.01 |
| Self-control | **0.21** | **0.18** | **0.23** | -0.04 | -0.07 | 0.00 | -0.01 | -0.01 | 0.00 |
| Consideration of future consequences | **0.07** | **0.04** | **0.09** | 0.00 | -0.02 | 0.03 | 0.00 | 0.00 | 0.00 |
| Risk-taking | **0.16** | **0.13** | **0.18** | **0.06** | **0.03** | **0.08** | **0.01** | **0.01** | **0.01** |
| Anxiety | **0.23** | **0.21** | **0.26** | 0.05 | 0.01 | 0.09 | 0.01 | 0.00 | 0.02 |
| Peer problems | **0.22** | **0.20** | **0.24** | **0.12** | **0.09** | **0.14** | **0.03** | **0.02** | **0.03** |
| Hyperactivity | **0.23** | **0.20** | **0.25** | 0.01 | -0.03 | 0.04 | 0.00 | -0.01 | 0.01 |
| Conduct problems | **0.22** | **0.20** | **0.25** | **0.10** | **0.07** | **0.13** | **0.02** | **0.02** | **0.03** |
| Depressive symptoms | **0.27** | **0.25** | **0.29** | 0.01 | -0.02 | 0.05 | 0.00 | -0.01 | 0.01 |
| Aggression | **0.22** | **0.20** | **0.24** | **0.07** | **0.04** | **0.10** | **0.02** | **0.01** | **0.02** |
| Antisocial behavior | **0.19** | **0.17** | **0.22** | 0.01 | -0.02 | 0.03 | 0.00 | 0.00 | 0.01 |
| Attention-deficit hyperactivity disorder | **0.26** | **0.24** | **0.29** | 0.04 | 0.01 | 0.06 | 0.01 | 0.00 | 0.02 |
| Psychotic experiences | **0.40** | **0.38** | **0.43** | **0.07** | **0.04** | **0.09** | **0.03** | **0.02** | **0.04** |
| Subjective well-being | **0.21** | **0.20** | **0.24** | **0.10** | **0.08** | **0.13** | **0.02** | **0.02** | **0.03** |
| Model 2 |  |  |  |  |  |  |  |  |  |
| Negative/disordered affect | **0.36** | **0.33** | **0.39** | **0.19** | **0.16** | **0.22** | **0.07** | **0.06** | **0.08** |
| Externalizing tendencies | **0.33** | **0.30** | **0.35** | **0.14** | **0.10** | **0.17** | **0.04** | **0.03** | **0.06** |

*Note*. Standardized estimates. *a*=effect of childhood maltreatment on the candidate mediator; *b*=effect of the candidate mediator on intimate partner violence (IPV) victimization; *ab*=indirect effect of maltreatment on IPV victimization via the mediator; lCI = 95% confidence intervals, lower bound; uCI = 95% confidence intervals, upper bound. Model 1 considered all intermediary phenotypes; Model 2 additionally estimated two latent factors accounting for their covariation as mediators. In Model 1, the total effect of childhood maltreatment on IPV victimization was *c*=0.24 (0.21, 0.26), the total indirect effect was ∑*ab*=0.14 (0.12, 0.15; 58% mediated), and the direct effect controlling for all mediators was *c'*=0.10 (0.07, 0.12). In Model 2, the total effect was *c*=0.24 (0.21, 0.26), the total indirect effect was ∑*ab*=0.11 (0.10, 0.12; 48% mediated), and the direct effect controlling for the two latent factors was *c'*=0.12 (0.10, 0.15). Statistically significant estimates (*p__adj_* < 0.003) are highlighted in bold font.

#### Supplementary Table 19 (ST19). Model fit indices, MR-DoC models

| Model | Mediator | Parameters | *df* | Fit (−2lnL) | AIC | BIC |
| --- | --- | --- | --- | --- | --- | --- |
| Baseline | - | 15 | 57,095 | 155,648.40 | 155,678.50 | 155,740.80 |
| Simple mediation | Neuroticism | 23 | 79,771 | 217,544.60 | 217,590.70 | 217,686.20 |
|  | Extraversion | 23 | 79,771 | 218,595.00 | 218,641.10 | 218,736.70 |
|  | Agreeableness | 23 | 79,771 | 219,333.10 | 219,379.20 | 219,474.80 |
|  | Conscientiousness | 23 | 79,771 | 218,882.00 | 218,928.10 | 219,023.70 |
|  | Self-control | 23 | 79,771 | 217,718.50 | 217,764.60 | 217,860.20 |
|  | Consideration of future consequences | 23 | 79,771 | 219,254.00 | 219,300.10 | 219,395.60 |
|  | Risk-taking | 23 | 79,771 | 217,601.20 | 217,647.30 | 217,742.80 |
|  | Anxiety | 26 | 79,768 | 217,168.60 | 217,220.80 | 217,328.80 |
|  | Peer problems | 26 | 79,768 | 216,892.70 | 216,944.80 | 217,052.80 |
|  | Hyperactivity | 23 | 79,771 | 217,585.60 | 217,631.70 | 217,727.20 |
|  | Conduct problems | 23 | 79,771 | 217,615.60 | 217,661.70 | 217,757.30 |
|  | Depression | 23 | 79,771 | 216,534.00 | 216,580.10 | 216,675.60 |
|  | Aggression | 23 | 79,771 | 217,824.20 | 217,870.30 | 217,965.80 |
|  | Antisocial behavior | 23 | 79,771 | 218,410.80 | 218,456.90 | 218,552.40 |
|  | ADHD | 23 | 79,771 | 216,542.80 | 216,588.90 | 216,684.40 |
|  | Psychotic experiences | 23 | 79,771 | 214,074.10 | 214,120.20 | 214,215.80 |
|  | Wellbeing | 23 | 79,771 | 217,598.80 | 217,644.90 | 217,740.40 |
| Model 3 | Psychotic experiences, peer problems, well-being, aggression, conduct problems, and risk-taking | 75 | 193,139 | 514,293.30 | 514,444.30 | 514,755.20 |
| Model 4 | Negative/disordered affect, externalizing tendencies | 97 | 465,325 | 1,192,973.00 | 1,193,168.00 | 1,193,570.00 |

*Note*. Df = Degrees of freedom;−2LL = −2 log-likelihood; AIC = sample-size adjusted Akaike Information Criterion, BIC = sample-size adjusted Bayesian Information Criterion.

# References

1. Houtepen, L. C., Heron, J., Suderman, M. J., Tilling, K. & Howe, L. D. Adverse childhood experiences in the children of the avon longitudinal study of parents and children (ALSPAC). *Wellcome Open Res* **3**, (2018).

2. Black, M. C. *et al.* *National Intimate Partner and Sexual Violence Survey: 2010 Summary Report*. (2011).

3. Mullins-Sweatt, S. N., Jamerson, J. E., Samuel, D. B., Olson, D. R. & Widiger, T. A. Psychometric properties of an abbreviated instrument of the five-factor model. *Assessment* **13**, 119–137 (2006).

4. Tangney, J. P., Boone, A. L. & Baumeister, R. F. High self-control predicts good adjustment, less pathology, better grades, and interpersonal success. in *Self-regulation and self-control* 173–212 (Routledge, 2018).

5. Strathman, A., Gleicher, F., Boninger, D. S. & Edwards, C. S. The consideration of future consequences: Weighing immediate and distant outcomes of behavior. *J Pers Soc Psychol* **66**, 742 (1994).

6. Nicholson, N., Soane, E., Fenton‐O’Creevy, M. & Willman, P. Personality and domain‐specific risk taking. *J Risk Res* **8**, 157–176 (2005).

7. Goodman, R. The strengths and difficulties questionnaire: A research note. *J Child Psychol Psychiatry* **38**, 581–586 (1997).

8. Messer, S. C. *et al.* Development of a short questionnaire for use in epidemiological studies of depression in children and adolescents: Factor composition and structure across development. *Int J Methods Psychiatr Res* **5**, 251–262 (1995).

9. Webster, G. D. *et al.* The brief aggression questionnaire: Psychometric and behavioral evidence for an efficient measure of trait aggression. *Aggress Behav* **40**, 120–139 (2014).

10. McAra, L. & McVie, S. Youth crime and justice: Key messages from the Edinburgh Study of Youth Transitions and Crime. *Criminology & Criminal Justice* **10**, 179–209 (2010).

11. Conners, C. K. Conners third edition (Conners 3). *Los Angeles, CA: Western Psychological Services* (2008).

12. Ronald, A. *et al.* Characterization of psychotic experiences in adolescence using the specific psychotic experiences questionnaire: findings from a study of 5000 16-year-old twins. *Schizophr Bull* **40**, 868–877 (2014).

13. Lavallee, L. F., Hatch, P. M., Michalos, A. C. & McKinley, T. Development of the contentment with life assessment scale (CLAS): Using daily life experiences to verify levels of self-reported life satisfaction. *Soc Indic Res* **83**, 201–244 (2007).

14. Phillips, A. R. *et al.* Lived-experience perspectives on the psychological factors linking childhood maltreatment to later intimate partner violence victimization. *Eur J Psychotraumatol* (2025).

15. R Core Team. R: A language and environment for statistical computing. Preprint at (2022).

16. Wickham, H., François, R., Henry, L. & Müller, K. Vaughan, D. dplyr: A grammar of data manipulation. *R package version* **1**, (2023).

17. Revelle, W. psych: Procedures for psychological, psychometric, and personality research. Preprint at (2022).

18. Tierney, N. J. & Cook, D. H. Expanding tidy data principles to facilitate missing data exploration, visualization and assessment of imputations. *arXiv preprint arXiv:1809.02264* (2018).

19. Van Buuren, S. & Groothuis-Oudshoorn, K. mice: Multivariate imputation by chained equations in R. *J Stat Softw* **45**, 1–67 (2011).

20. Liang, K.-Y. & Zeger, S. L. Longitudinal data analysis using generalized linear models. *Biometrika* **73**, 13–22 (1986).

21. Kline, R. B. *Principles and Practice of Structural Equation Modeling*. (Guilford publications, 2023).

22. Li, C. Little’s test of missing completely at random. *Stata J* **13**, 795–809 (2013).

23. White, I. R., Royston, P. & Wood, A. M. Multiple imputation using chained equations: issues and guidance for practice. *Stat Med* **30**, 377–399 (2011).

24. McDonald, R. P. *Test Theory: A Unified Treatment*. *Test Theory: A Unified Treatment* (Taylor and Francis, 2013). doi:10.4324/9781410601087.

25. Shrout, P. E. & Fleiss, J. L. Intraclass correlations: uses in assessing rater reliability. *Psychol Bull* **86**, 420 (1979).

26. Rosseel, Y. lavaan: An R package for structural equation modeling. *J Stat Softw* **48**, 1–36 (2012).

27. Højsgaard, S., Halekoh, U. & Yan, J. The R package geepack for generalized estimating equations. *J Stat Softw* **15**, 1–11 (2006).

28. Bonferroni, C. Teoria statistica delle classi e calcolo delle probabilita. *Pubblicazioni del R istituto superiore di scienze economiche e commericiali di firenze* **8**, 3–62 (1936).

29. Warrier, V. *et al.* Gene–environment correlations and causal effects of childhood maltreatment on physical and mental health: a genetically informed approach. *Lancet Psychiatry* **8**, 373–386 (2021).

30. Nagel, M. *et al.* Meta-analysis of genome-wide association studies for neuroticism in 449,484 individuals identifies novel genetic loci and pathways. *Nat Genet* **50**, 920–927 (2018).

31. De Moor, M. H. M. *et al.* Meta-analysis of genome-wide association studies for personality. *Mol Psychiatry* **17**, 337–349 (2012).

32. Karlsson Linnér, R. *et al.* Genome-wide association analyses of risk tolerance and risky behaviors in over 1 million individuals identify hundreds of loci and shared genetic influences. *Nat Genet* **51**, 245–257 (2019).

33. Purves, K. L. *et al.* A major role for common genetic variation in anxiety disorders. *Mol Psychiatry* **25**, 3292–3303 (2020).

34. McIntosh, A. M., Lewis, C. M., Adams, M. J. & Group, P. G. C. M. D. D. W. Genome-wide study of half a million individuals with major depression identifies 697 independent associations, infers causal neuronal subtypes and biological targets for novel pharmacotherapies. *medRxiv* (2024).

35. Ip, H. F. *et al.* Genetic association study of childhood aggression across raters, instruments, and age. *Transl Psychiatry* **11**, 413 (2021).

36. Karlsson Linnér, R. *et al.* Multivariate analysis of 1.5 million people identifies genetic associations with traits related to self-regulation and addiction. *Nat Neurosci* **24**, 1367–1376 (2021).

37. Williams, C. M. *et al.* Guidelines for evaluating the comparability of down-sampled GWAS summary statistics. *Behav Genet* **53**, 404–415 (2023).

38. Trubetskoy, V. *et al.* Mapping genomic loci implicates genes and synaptic biology in schizophrenia. *Nature* **604**, 502–508 (2022).

39. Demontis, D. *et al.* Genome-wide analyses of ADHD identify 27 risk loci, refine the genetic architecture and implicate several cognitive domains. *Nat Genet* **55**, 198–208 (2023).

40. Okbay, A. *et al.* Genetic variants associated with subjective well-being, depressive symptoms, and neuroticism identified through genome-wide analyses. *Nat Genet* **48**, 624–633 (2016).

41. Staiger, D. O. & Stock, J. H. *Instrumental Variables Regression with Weak Instruments*. (1994).

42. Tackett, J. L., Quilty, L. C., Sellbom, M., Rector, N. A. & Bagby, R. M. Additional evidence for a quantitative hierarchical model of mood and anxiety disorders for DSM-V: the context of personality structure. *J Abnorm Psychol* **117**, 812 (2008).

43. Samuel, D. B. & Widiger, T. A. A meta-analytic review of the relationships between the five-factor model and DSM-IV-TR personality disorders: A facet level analysis. *Clin Psychol Rev* **28**, 1326–1342 (2008).

44. van Prooijen, J.-W. & van der Kloot, W. A. Confirmatory analysis of exploratively obtained factor structures. *Educ Psychol Meas* **61**, 777–792 (2001).

45. Asparouhov, T. & Muthén, B. Exploratory structural equation modeling. *Struct Equ Modeling* **16**, 397–438 (2009).

46. Neale, M. C. & Cardon, L. *Methodology for Genetic Studies of Twins and Families*. (Springer Science & Business Media, 2013). doi:10.1136/jmg.30.9.800-a.

47. Voronin, I. TwinAnalysis: This is a package to simplify structural equation modeling - and particularly, twin analysis - in R. Preprint at (2022).

48. Bulik-Sullivan, B. *et al.* An atlas of genetic correlations across human diseases and traits. *Nat Genet* **47**, 1236–1241 (2015).

49. Consortium, B. *et al.* Analysis of shared heritability in common disorders of the brain. *Science (1979)* **360**, eaap8757 (2018).

50. Tielbeek, J. J. *et al.* Uncovering the genetic architecture of broad antisocial behavior through a genome-wide association study meta-analysis. *Mol Psychiatry* **27**, 4453–4463 (2022).

51. Choi, S. W., Mak, T. S.-H. & O’Reilly, P. F. Tutorial: a guide to performing polygenic risk score analyses. *Nat Protoc* **15**, 2759–2772 (2020).

52. Bulik-Sullivan, B. K. *et al.* LD Score regression distinguishes confounding from polygenicity in genome-wide association studies. *Nat Genet* **47**, 291–295 (2015).

53. Grotzinger, A. D. *et al.* Genomic structural equation modelling provides insights into the multivariate genetic architecture of complex traits. *Nat Hum Behav* **3**, 513–525 (2019).

54. Benjamini, Y. & Hochberg, Y. Controlling the false discovery rate: a practical and powerful approach to multiple testing. *Journal of the Royal statistical society: series B (Methodological)* **57**, 289–300 (1995).

55. R Core Team. R: A language and environment for statistical computing. Preprint at https://www.r-project.org/ (2022).

56. Vilhjálmsson, B. J. *et al.* Modeling linkage disequilibrium increases accuracy of polygenic risk scores. *The american journal of human genetics* **97**, 576–592 (2015).

57. Selzam, S. *et al.* Comparing within-and between-family polygenic score prediction. *The American Journal of Human Genetics* **105**, 351–363 (2019).

58. Minică, C. C., Dolan, C. V, Boomsma, D. I., de Geus, E. & Neale, M. C. Extending causality tests with genetic instruments: an integration of Mendelian randomization with the classical twin design. *Behav Genet* **48**, 337–349 (2018).

59. Neale, M. C. *et al.* OpenMx 2.0: Extended structural equation and statistical modeling. *Psychometrika* **81**, 535–549 (2016).

60. Smith, G. D. & Ebrahim, S. ‘Mendelian randomization’: Can genetic epidemiology contribute to understanding environmental determinants of disease? *Int J Epidemiol* **32**, 1–22 (2003).

61. Burgess, S. & Thompson, S. G. *Mendelian Randomization: Methods for Using Genetic Variants in Causal Estimation*. (CRC Press, 2015).

62. Gillespie, N. A. & Martin, N. G. Direction of causation models. *Encyclopedia of statistics in behavioral science* (2005).

63. Duffy, D. L. & Martin, N. G. Inferring the direction of causation in cross‐sectional twin data: Theoretical and empirical considerations. *Genet Epidemiol* **11**, 483–502 (1994).

64. Oginni, O. A. *et al.* Bidirectional causal associations between same-sex attraction and psychological distress: Testing moderation and mediation effects. *Behav Genet* **53**, 118–131 (2023).
